# Investigation of Antibody Tolerance in Methanol for Analytical Purposes: Methanol Effect Patterns and Molecular Mechanisms

**DOI:** 10.1002/advs.202402050

**Published:** 2024-06-18

**Authors:** Yingjie Zhang, Jiafei Mi, Weilin Wu, Jie Fei, Bochen Lv, Xuezhi Yu, Kai Wen, Jianzhong Shen, Zhanhui Wang

**Affiliations:** ^1^ National Key Laboratory of Veterinary Public Health and Safety Beijing Key Laboratory of Detection Technology for Animal‐Derived Food College of Veterinary Medicine China Agricultural University Beijing 100193 P. R. China

**Keywords:** antibody methanol effect patterns, antibody tolerance, folding and aggregation, immunoassay, molecular mechanism

## Abstract

The extraction of targets from biological samples for immunoassays using organic solvents, such as methanol, is often necessary. However, high concentrations of organic solvents in extracts invariably lead to instability of the employed antibody, resulting in poor performance of the immunoassay. Evaluating the tolerance ability and exploring the molecular mechanisms of antibody tolerance in organic solvents are essential for the development of robust immunoassays. In this work, 25 monoclonal antibodies and methanol are utilized as models to address these questions. A novel protocol is initially established to precisely and rapidly determine antibody tolerance in methanol, identifying two distinct methanol effect patterns. Through a detailed investigation of the structural basis, a novel hypothesis regarding methanol effect patterns is proposed, termed “folding‐aggregation,” which is subsequently validated through molecular dynamics simulations. Furthermore, the investigation of sequence basis reveals significant differences in residue types within the complementarity‐determining regions and ligand‐binding residues, distinguishing the two antibody methanol effect patterns. Moreover, the methanol effect patterns of the antibodies are defined by germline antibodies. This work represents the first exploration of antibody methanol effect patterns and associated molecular mechanisms, with potential implications for the discovery and engineering of tolerant antibodies for the development of robust immunoassays.

## Graphical abstract

This study provides insight into the methanol instability of antibodies in immunoassays. Using 25 monoclonal antibodies, a novel protocol assesses methanol tolerance, identifying two distinct patterns. Structural analysis proposes a “folding‐aggregation” hypothesis, validated by molecular dynamics simulations. Sequence analysis reveals differences in key residues, defining methanol effect patterns. Germline antibodies dictate these patterns, offering insights for robust immunoassay development.

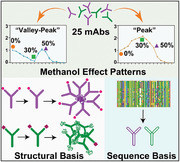

## 1 Introduction

Based on specific antibody‐antigen recognition, immunoassays are rapid, cost‐effective, highly sensitive, and specific tools in various fields including biomedical research, drug discovery, environment monitoring, food analysis, and security defense.^[^ ^1^, ^2^ ^]^ Typically, the analytical procedures involve extracting targets from biological and environmental samples into organic solvents, primarily methanol, to ensure the high accuracy of immunoassay.^[^ ^3^ ^]^ However, the organic solvents introduced from these extracts invariably compromise the stability and activity of antibodies, thereby affecting immunoassay performance in terms of sensitivity, accuracy, and precision.^[^ ^4^ ^]^ Consequently, obtained extracts are often either diluted with aqueous solutions, leading to compromised sensitivity, or subjected to clean‐up steps such as nitrogen blowing or immunoaffinity purification,^[^ ^5^, ^6^ ^]^ which extend assay times. Balancing analytical performance and efficiency, conducting immunoassays in the presence of high concentrations of organic solvents emerges as an attractive and highly demanded strategy. Nonetheless, immunoassays are predominantly carried out in aqueous solutions or gentle environments resembling biological fluids due to antibodies commonly being produced under physiological conditions intolerant to high concentrations of organic solvent.^[^ ^7^ ^]^ While some researchers have assessed antibody tolerance to various organic solvents and endeavored to develop immunoassays in their presence, their efforts have focused on selecting suitable solvents and dilution times for specific matrices.^[^ ^8^, ^9^, ^10^, ^11^, ^12^, ^13^, ^14^ ^]^ Regrettably, substantial dilutions (2–100 times) are typically necessary, resulting in sensitivity losses that may not meet regulatory requirements in some instances. Consequently, antibody tolerance to organic solvents remains a critical concern in immunoassay fields, necessitating the development of tolerant antibodies in organic solvent environments, a promising and practical endeavor.

To date, two primary strategies have been employed for the development of antibodies capable of tolerating organic solvents. The first involves a stress screening approach, wherein tolerant antibodies are identified through screening processes conducted with hybridomas or mutant libraries in the presence of organic solvents.^[^ ^5^, ^15^ ^]^ The second strategy entails antibody engineering techniques, such as complementarity‐determining regions (CDR) grafting and PEGylation.^[^ ^16^, ^17^ ^]^ However, both of these strategies currently exhibit limitations in terms of efficacy and efficiency, resulting in the acquisition of only a limited number of antibodies with significant tolerance levels suitable for practical application in immunoassays conducted in the presence of organic solvents. This limitation stems from the insufficient understanding of the mechanisms underlying antibody tolerance to organic solvents, which hampers the development of effective and rational approaches for antibody screening or engineering tailored for analytical purposes in organic solvent environments.

The properties of organic solvents have been demonstrated to influence the binding activity of antibodies. Binding activity between antibodies and hapten molecules in pure organic solvents (including dioxane, acetonitrile, 1‐propanol, 1‐butanol, and 1‐pentanol) was assessed, revealing a correlation between binding activity and solvent hydrophobicity.^[^ ^18^ ^]^ Specifically, the more hydrophobic the solvent, the weaker the antibody‐hapten interaction. Furthermore, the impact of four nonpolar organic solvents (dichloromethane, trichloromethane, toluene, and n‐hexane) on the binding activity of triazine and atrazine haptens with their antibodies was investigated.^[^ ^19^ ^]^ The binding activity of antibodies exhibited a correlation with solvent polarities and the solubility of triazine and its haptens in the solvents. Additionally, the influence of water‐miscible organic solvents on the binding activity of 2,4‐dichlorophenoxyacetic acid and 4,4′‐dichlorobiphenyl with corresponding antibodies was examined.^[^ ^20^ ^]^ The results showed that higher hydrophobicity (as indicated by logP values) of organic solvents resulted in increased antibody affinity, as reflected by higher values of association equilibrium constants. Despite the correlation between certain properties of organic solvents and antibody binding activity, the intrinsic molecular mechanisms underlying antibody tolerance in organic solvents remain inadequately studied.

The molecular mechanism of protein tolerance in organic solvents has been studied in the field of enzymes.^[^ ^21^, ^22^ ^]^ Typically, in an aqueous solvent, the polar groups of enzymes tend to reside on the surface, interacting with water molecules, while the hydrophobic groups are buried internally to form a hydrophobic core. The structural integrity of enzymes relies on a delicate balance of non‐covalent interactions, including hydrogen bonds, hydrophobic interactions, electrostatic charge interactions, and Van der Waals forces.^[^ ^23^ ^]^ However, in a medium containing organic solvents, enzyme deactivation often occurs due to the disruption of this hydrophobic core caused by changes in the medium's hydrophobicity.^[^ ^24^ ^]^ Additionally, hydrophobic patches present in the folding/unfolding intermediates of enzymes can initiate the aggregation process, leading to the formation of soluble oligomers and agglomerated aggregates.^[^ ^25^ ^]^ Similarly, the interaction between antibodies and ligands also relies on non‐covalent bonds, which are susceptible to disruption by organic solvents, akin to enzymes. Therefore, comprehending the structural changes of antibodies in organic solvents is crucial for elucidating their tolerance mechanisms.

In addition to comprehending the structural basis of antibodies in organic solvents, understanding the sequence basis is equally crucial, with the latter holding greater practical significance in the discovery and engineering of tolerant antibodies. The chemical properties of the functional groups present in each amino acid have been shown to contribute significantly to protein stability.^[^ ^26^ ^]^ Furthermore, research has indicated that the sequence characteristics of variable regions, particularly the complementarity‐determining region (CDR) loops, play a pivotal role in determining the physicochemical properties of antibodies.^[^ ^27^, ^28^ ^]^ Therefore, sequence analysis holds promise in elucidating the tolerance mechanisms of antibodies in organic solvents.

In this study, 25 antibodies targeting non‐steroidal anti‐inflammatory drugs (NSAIDs) served as models for investigating antibody tolerance in methanol. Initially, the binding activity of antibodies in methanol at various concentrations was assessed, leading to the identification of distinct methanol effect patterns. Subsequently, we conducted a comprehensive exploration of the structural basis underlying these different methanol effect patterns using a range of spectroscopic methods. A novel hypothesis termed “folding‐aggregation” was proposed for the first time and subsequently validated through molecular dynamics (MD) simulations. Furthermore, we elucidated the sequence basis of methanol effect patterns, offering potential insights for antibody discovery and engineering aimed at enhancing antibody tolerance and improving immunoassay performance in methanol.

## 2 Results and Discussion

### 2.1 The Measurements of Antibody Tolerance in Methanol

The tolerance of antibodies to organic solvents represents a critical challenge in immunoassay development, which could address the fundamental trade‐off between detection performance and assay duration. In this study, to investigate the tolerance of antibodies in organic solvents, 25 antibodies were used as model antibodies. The tolerance of these antibodies, as indicated by their binding activities with respective coating antigens, was assessed across a spectrum of methanol concentrations, ranging from 0% to 100%, utilizing the ncELISA, a widely employed immunoassay format.

**Figure 1:**
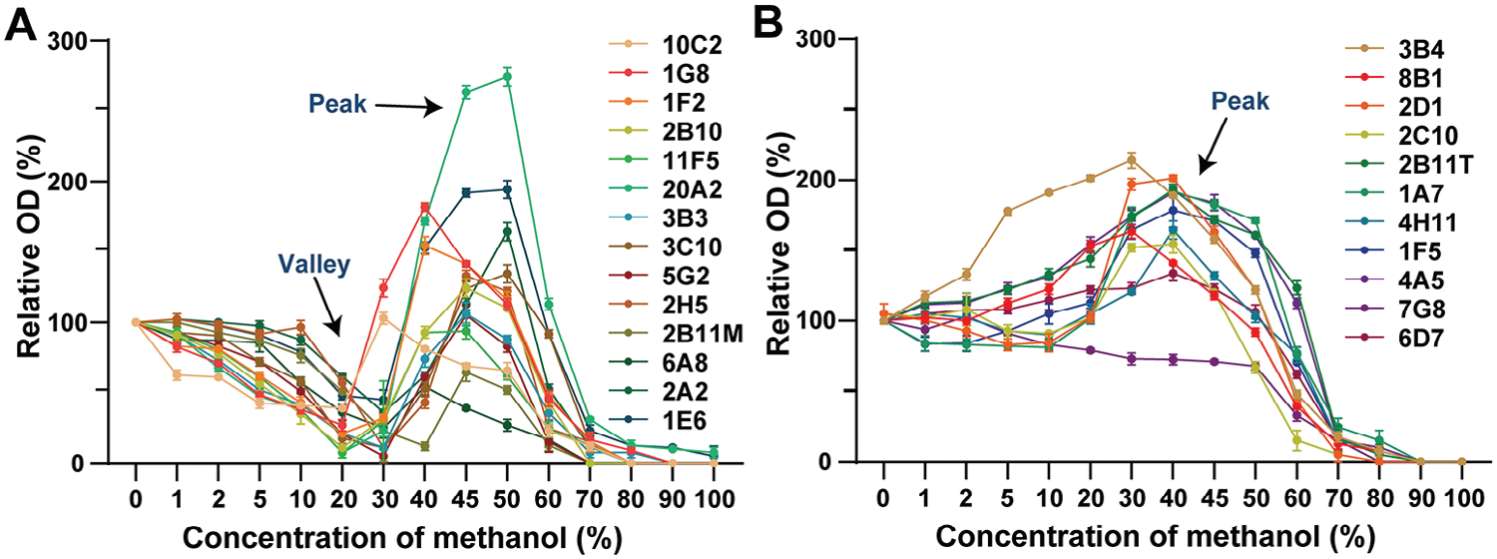
Methanol effect patterns in antibody tolerance. A) “Valley‐Peak” pattern curves demonstrate decreases, followed by increases, then decreases with rising methanol concentrations. B) “Peak” pattern curves show increases followed by decreases as methanol concentrations rise. The binding activity of antibodies to coating antigens in methanol were measured by ncELISA (N = 3).

To validate the methodology for assessing antibody tolerance in methanol, we initially examined the impact of methanol on the coating and blocking systems of the ncELISA, as detailed in the supporting information. Across concentrations ranging from 0% to 100%, methanol demonstrated no significant influence on the performance of these systems, nor did it induce non‐specific adsorption of the primary antibodies and second antibody to the coating antigens and OVA carrier (Figure S2, Supporting Information). Consequently, the binding activity of antibodies to coating antigens in methanol was accurately measured and represented by relative OD values against methanol concentration, delineating the tolerance curves of antibodies. Lower relative OD values indicated diminished binding activities of antibodies to coating antigens in methanol. Remarkably, the tolerance curves of the 25 antibodies exhibited nonlinearity and distinctive regularities, effectively classifiable into two methanol effect patterns (**Figure** **1**A,B). The first pattern, termed the “Valley‐Peak” pattern, showcased declines followed by increases, then subsequent declines with escalating methanol concentrations, characterized by a discernible valley and peak (Figure 1A). Conversely, the second pattern, termed the “Peak” pattern, displayed increases followed by declines with rising methanol concentrations, denoted solely by a peak (Figure 1B). Of the tested antibodies, 14 belonged to the “Valley‐Peak” pattern (Pattern 1), while the remaining 11 belonged to the “Peak” pattern (Pattern 2).

At methanol concentrations below 30%, Pattern 1 antibodies exhibited a consistent decline in binding activity, with curves reaching their valleys at ≈30% methanol. In contrast, Pattern 2 antibodies displayed a steady increase in binding activity, with curves reaching their peaks at ≈30% methanol. Between methanol concentrations of 30% and 50%, Pattern 1 antibodies experienced a temporary increase in binding activity, peaking at ≈50% methanol. Conversely, the binding activities of Pattern 2 antibodies rapidly decreased during this range, reaching values equivalent to those in 0% methanol. At methanol concentrations exceeding 50%, both antibody patterns demonstrated sharp declines, with binding nearly abolished at concentrations above 80%. This suggests that high concentrations of methanol can severely impair the binding between antibody and antigen.

This study represents the first comprehensive examination of the impact of methanol on antibodies for the purpose of immunoassay development, revealing significant regularities in the tolerance curves of antibodies in methanol. To validate the universality of our findings, we conducted a thorough analysis and comparison of reported tolerance curves from studies where the final methanol concentration exceeded 40%. As shown in Table S2 (Supporting Information), these two distinct methanol effect patterns appear to be widespread among antibodies derived from mice, rabbits, and camels, despite the limited availability of antibody tolerance data from these studies.^[^ ^8^, ^9^, ^11^, ^29^ ^]^ The ubiquity and consistency of these effect patterns in organic solvents have not been previously documented or summarized, likely due to the absence of large‐scale and detailed investigations into antibody tolerance in methanol. However, the precise reasons behind the manifestation of such regular tolerance curves by antibodies, as well as the underlying molecular mechanisms, remain unclear.

### 2.2 The Measurement of Antibodies Secondary Structure in Methanol

To delve into the intrinsic mechanisms underlying the two methanol effect patterns, we investigated the structural basis of antibodies in methanol. Initially, CD spectroscopy in the far UV region was employed to explore potential alterations in the secondary structure, focusing on elements such as α‐helix, β‐sheet, β‐turn, and random coil structures.^[^ ^30^ ^]^ As shown in Figure 1A,B, the observed variations in the tolerance curves of antibodies suggested the likelihood of conformational changes in their secondary structures. Notably, MAA‐specific antibodies and TLF‐specific antibodies constituted the predominant categories within Pattern 1 and Pattern 2, respectively. Consequently, we selected the MAA‐specific antibody 3B3 and the TLF‐specific antibody 3B4 as model antibodies for Pattern 1 and Pattern 2, respectively, and proceeded to assess the secondary structures of 3B3 and 3B4 using CD spectroscopy across varying concentrations of methanol.

**Figure 2:**
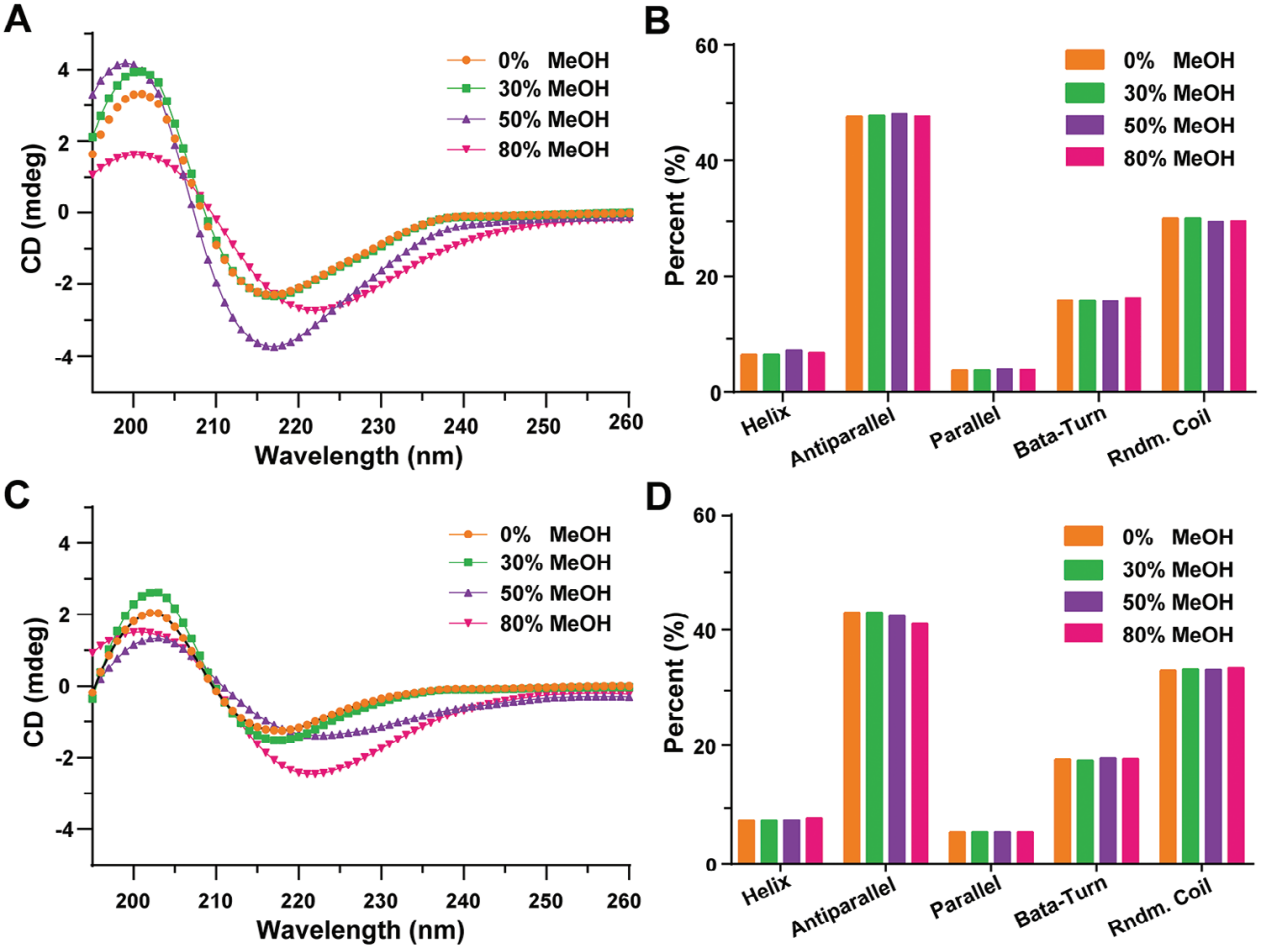
Antibody secondary structure analysis in methanol. A) CD spectrum and B) percentage of each secondary structure for 3B3 in 0%, 30%, 50%, and 80% methanol. C) CD spectrum and D) percentage of each secondary structure for 3B4 in 0%, 30%, 50%, and 80% methanol.

As shown in **Figure** **2**A,B, the CD spectra of 3B3 across varying concentrations of methanol (0%, 30%, 50%, and 80%) revealed a consistent distribution of secondary structure components. This suggests that the types and proportions of α‐helix, antiparallel β‐sheet, parallel β‐sheet, β‐turn, and random coil structures within 3B3 remained largely unchanged with increasing methanol concentrations. Similarly, as illustrated in Figure 2C,D, the CD spectra of 3B4 under the same methanol concentrations exhibited a comparable distribution of secondary structures to those observed in 3B3. This indicates that the types and proportions of secondary structures remained relatively stable across varying methanol concentrations for both antibodies. The CD analysis suggests that that methanol did not significantly alter the secondary structures of antibodies in either pattern. However, notable differences were observed in the signal intensities of the CD spectra for 3B3 and 3B4 across different methanol concentrations, implying potential alterations in the spatial structures of these antibodies in response to varying methanol concentrations.^[^ ^31^ ^]^ Further investigation into these structural changes in methanol is warranted.

### 2.3 The Measurements of Antibodies Folding and Aggregation Behaviors in Methanol

To further investigate the structural basis of the two antibody patterns, we examined the folding and aggregation behaviors of antibodies in methanol. The folding behavior of 3B3 and 3B4 in methanol concentrations of 0%, 30%, and 50% was analyzed using DSF to measure the intrinsic fluorescence intensity of tryptophan. The BCM of the fluorescence emission spectra against temperature was plotted to assess folding behavior. Additionally, the aggregation behavior of 3B3 and 3B4 was monitored using SLS, and the scattered signal value at 266 nm was plotted. These measurements were conducted over a temperature range of 25 to 42 °C, which is commonly used in immunoassay procedures.

**Figure 3:**
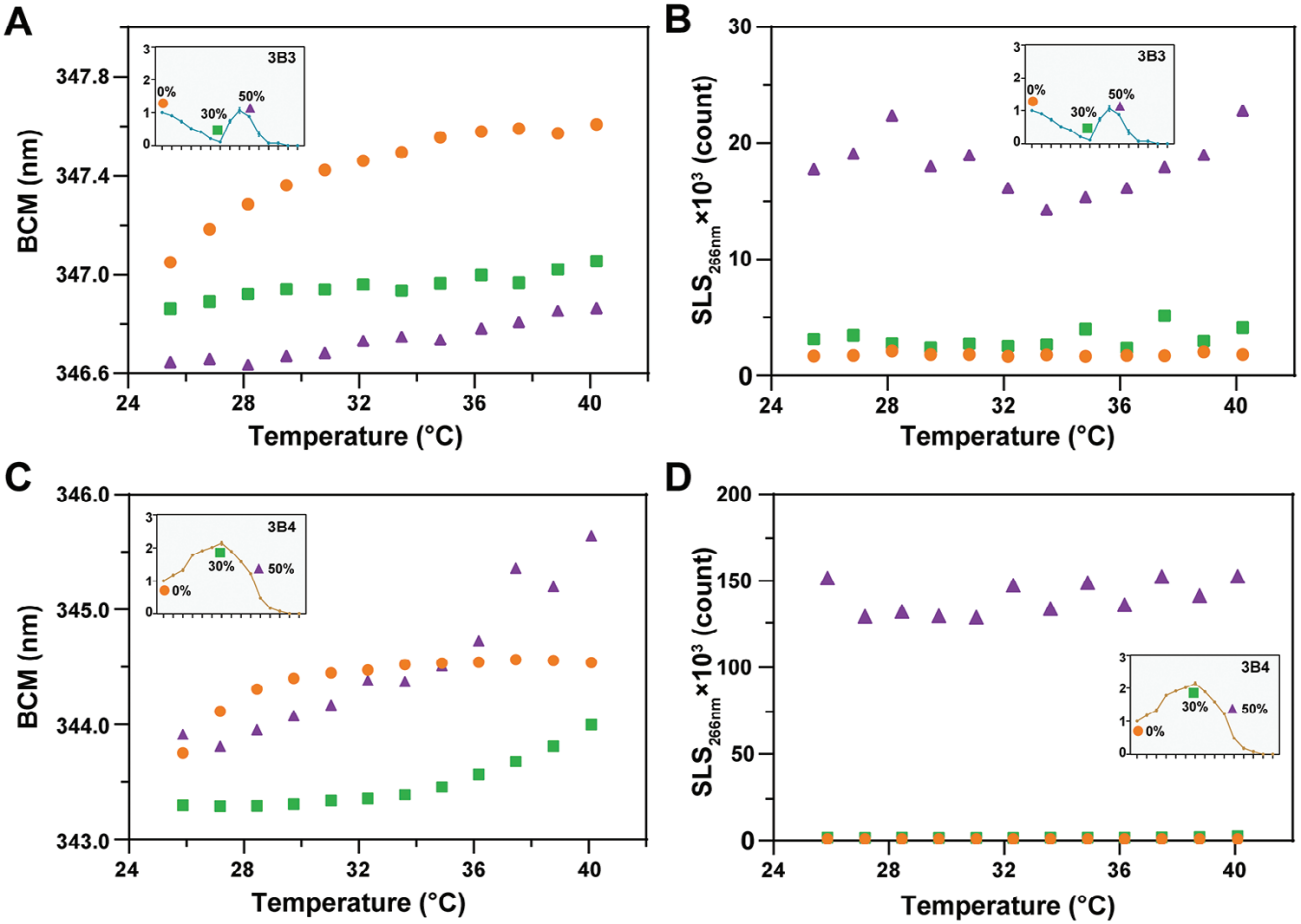
Folding and aggregation behavior analysis of 3B3 and 3B4 in methanol. A,B) DSF and SLS analysis of 3B3 in 0%, 30%, and 50% methanol. C,D) DSF and SLS analysis of 3B4 in 0%, 30% and 50% methanol. Yellow dots, green squares, and purple triangles represent the folding and aggregation behavior of the antibody in 0% methanol, 30% methanol, and 50% methanol, respectively.

In 30% methanol, the tolerance curve of 3B3 decreases to its lowest level, indicating significant damage to the binding activity of 3B3 (Figure 1A). As shown in **Figure** **3**A, 3B3 exhibited significantly decreased BCM values in 30% methanol compared to those in 0% methanol at each sampling point, suggesting that 3B3 adopts a more compact conformation in 30% methanol. Therefore, the folding behavior analysis revealed that methanol induced significant refolding of 3B3 as its concentration increased from 0% to 30%. Furthermore, as illustrated in Figure 3B, the SLS_266 nm_ values of 3B3 remained at lower levels both in 0% and 30% methanol, indicating that no significant aggregation occurred for 3B3 under these conditions. Consequently, the aggregation behavior analysis demonstrated that methanol had no notable impact on the aggregation behavior of 3B3 as its concentration increased from 0% to 30%. Combining the analyses of folding and aggregation behavior, it can be speculated that the refolding state of 3B3 in 30% methanol contributed to its lowest binding activity, possibly due to the compact conformation of 3B3 in 30% methanol disrupting the interactions between the antibody and antigen.

In 50% methanol, the tolerance curve of 3B3 approaches its highest value of binding activity (Figure 1A). At this condition, 3B3 exhibits slightly decreased BCM values compared to those in 30% methanol at each sampling point, indicating that 3B3 adopts a more compact conformation in 50% methanol (Figure 3A). Consequently, the folding behavior analysis reveals that methanol induces further refolding of 3B3 as its concentration increases from 30% to 50%. As shown in Figure 3B, the SLS_266 nm_ values of 3B3 significantly increase in 50% methanol, indicating the occurrence of noticeable aggregation. Thus, the aggregation behavior analysis demonstrates that methanol induces aggregation of 3B3 as its concentration increases from 30% to 50%. It can be inferred that the partially unfolded monomers of 3B3 in 50% methanol form soluble oligomers or agglomerated aggregates, possibly enhancing the avidity of the antibody for its antigen binding.^[^ ^32^ ^]^ Although the refolding of 3B3 remains unfavorable for binding activities in 50% methanol, the increased avidity of antibodies compensates for the loss of binding activity resulting from unfavorable refolding, leading to the formation of a binding activity peak ≈50% methanol. Beyond 50% methanol, the antibody undergoes irreversible precipitation as it forms large aggregates.^[^ ^33^ ^]^ Based on these results, the tolerance of 3B3 in methanol is associated with both the refolding and aggregation behavior of the antibody, revealing a novel finding that could be summarized as “folding unfavorable and aggregation favorable.”

For 3B4 of Pattern 2 antibodies, the tolerance curve reaches its highest level in 30% methanol and decreases by about half in 50% methanol compared to the peak values (Figure 1B). In 30% methanol, 3B4 exhibits decreased BCM values compared to those in 0% methanol at each sampling point, indicating that 3B4 adopts a more compact conformation in 30% methanol similar to 3B3 (Figure 3C). Consequently, the folding behavior analysis reveals that methanol induces refolding of 3B4 as its concentration increases from 0% to 30%. As shown in Figure 3D, the SLS_266 nm_ values of 3B4 remain at lower levels in both 0% and 30% methanol, indicating that no noticeable aggregation occurs for 3B4 in these conditions. Therefore, the aggregation behavior analysis shows that methanol has no significant influence on the aggregation behavior of 3B4 as its concentration increases from 0% to 30%, similar to 3B3. Combining the analysis of folding and aggregation behavior, we can speculate that the refolding state of 3B4 in 30% methanol benefits the binding activity compared to that of 3B3, possibly because the compact conformation of 3B4 in 30% methanol contributes to the interactions between the antibody and coating antigen.

In 50% methanol, 3B4 exhibits significantly increased BCM values compared to those in 30% methanol, particularly at sampling temperatures exceeding 36°C, indicating that methanol induces unfolding of 3B4 as its concentration increases from 30% to 50% (Figure 3C). In Figure 3D, the SLS_266 nm_ values of 3B4 show a significant increase, indicating the occurrence of noticeable aggregation for 3B4 in 50% methanol. These results suggest that the reduction in the binding activity of 3B4 in 50% methanol is primarily associated with the formation of aggregates, contrary to the behavior observed for 3B3 in Pattern 1 antibodies. The decrease in antibody binding activity due to antibody aggregates may be attributed to the orientation of antibody molecules during aggregate formation.^[^ ^32^ ^]^ When most of the variable regions of antibody molecules fold into aggregates, this aggregation significantly diminishes the opportunities for antibody‐antigen binding, ultimately reducing the binding activity of the antibody. Based on the aforementioned analysis, the binding activity of 3B4 in methanol is influenced by both unfolding and aggregation behaviors, a scenario that could be summarized as “folding favorable and aggregation unfavorable.” In light of our investigations into folding and aggregation behaviors, we propose the “folding‐aggregation” hypothesis to elucidate the two methanol effect patterns.

We also observed that both Pattern 1 and Pattern 2 antibodies exhibited increased BCM values as temperature increased in 0%, 30%, and 50% methanol, indicating typical thermal denaturation and unfolding (Figure 3A,C). However, this behavior was not reflected in the aggregation behavior of the antibodies, as there were no significant changes in SLS_266 nm_ values (Figure 3B,D).

Interestingly, the effects of methanol and temperature on antibody folding behavior could be used to differentiate between Pattern 1 and Pattern 2 antibodies. For 3B3 of Pattern 1 antibodies, the changes in BCM values from 25 to 42 °C in 0%, 30%, and 50% methanol were 0.57, 0.19, and 0.22 nm, respectively, indicating that the thermal stability of these antibodies increased in 30% and 50% methanol. This observation aligns with previous studies indicating that methanol enhances protein stability by significantly expanding the range of temperatures at which proteins remain stable.^[^ ^34^ ^]^

For 3B4 of Pattern 2 antibodies, the BCM value changes from 25 to 42 °C in 0%, 30%, and 50% methanol were 1.20, 0.71, and 1.72 nm, respectively, suggesting that thermal stability increased in 30% methanol but decreased in 50% methanol. In 30% methanol, the stabilizing effect of methanol on proteins seems dominant, while in 50% methanol, denaturing effects are more pronounced.

Additionally, we observed that Pattern 1 and Pattern 2 antibodies showed different responses to temperature in terms of methanol tolerance. For 3B3 of Pattern 1 antibodies, BCM values were consistently highest in 50% methanol and lowest in 0% methanol, suggesting that temperature did not significantly affect methanol tolerance. However, for 3B4 of Pattern 2 antibodies, BCM values for 0% methanol were higher than those for 50% methanol at temperatures below 37 °C, but lower at temperatures above 37 °C, indicating that temperature influences the methanol tolerance of this antibody.

Overall, Pattern 1 and Pattern 2 antibodies exhibited distinct behaviors in response to methanol's impact on their thermal stability and temperature's influence on their methanol tolerance.

### 2.4 The Validation of “Folding‐Aggregation” Hypothesis by MD Simulations

To validate the “folding‐aggregation” hypothesis, we conducted MD simulations of the 3B3 and 3B4 antibodies binding with their respective ligands in different concentrations of methanol. Firstly, we obtained the variable region sequences of 3B3 and 3B4, and the detailed methods are provided in the supporting information. Next, we constructed and verified the variable region structures of 3B3 and 3B4 using Ramachandran plots and Profile‐3D plots (Figure S3, Supporting Information). Subsequently, we prepared the complexes of 3B3‐MAA and 3B4‐TLF using the CDOCKER module of DS software (Figure S4, Supporting Information), followed by 100 ns MD simulations conducted using GROMACS in 0%, 30%, and 50% methanol.

**Figure 4:**
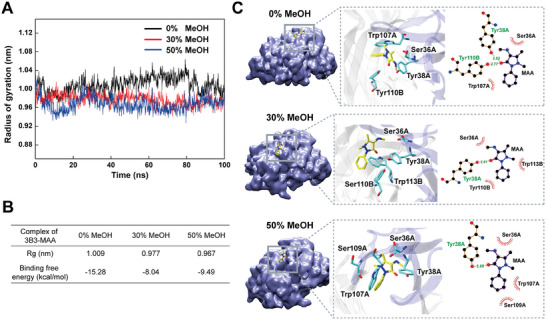
MD simulations of 3B3 with ligand MAA in methanol. A,B) Average gyration radius and binding free energy of 3B3 in 0%, 30%, and 50% methanol. C) Complex structure and 2D interactions of 3B3 with ligand MAA in 0%, 30%, and 50% methanol.

The radius of gyration serves as a measure to characterize the tightness of folding within the antibody binding cavity, with a smaller radius indicating a tighter folding.^[^ ^35^ ^]^ As shown in **Figure** **4**A,B, the average gyration radii of 3B3 residues within 0.6 nm of the ligand MAA were 1.009, 0.977, and 0.967 nm in 0%, 30%, and 50% methanol, respectively. These results suggest that the binding cavity of 3B3 underwent continuous refolding with increasing concentrations of methanol, consistent with the overall refolding process observed for 3B3 using DSF (Figure 2A).

Furthermore, the binding free energy of 3B3 to the MAA ligand was found to be −15.28, −8.04, and −9.49 kcal mol^−1^ in 0%, 30%, and 50% methanol, respectively (Figure 4B). This trend aligns with the observed in the tolerance curve of 3B3, where the binding activity initially decreased and then increased (Figure 1A). Additionally, interaction analysis revealed that in 0% methanol, the ligand MAA formed two hydrogen bonds with Tyr38A and Tyr110B of 3B3, whereas in 30% and 50% methanol, only one hydrogen bond formed with Tyr38A (Figure 4C). This suggests that methanol disrupted the interaction between 3B3 and the MAA ligand.

It is noteworthy that the comparable binding activities of 3B3 to MAA in 0% and 50% methanol may have resulted from a combination of decreased antibody affinity due to folding and increased antibody avidity due to aggregation. Therefore, the results of MD simulations support the classification of 3B3 as an antibody type exhibiting “folding unfavorable and aggregation favorable” characteristics, consistent with our proposed hypothesis.

**Figure 5:**
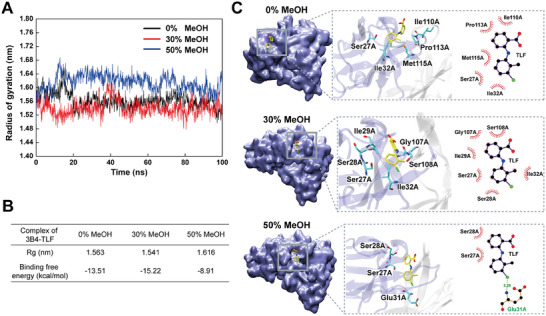
MD simulations of 3B4 with ligand TLF in methanol. A,B) Average gyration radius and binding free energy of 3B4 in 0%, 30%, and 50% methanol. C) Complex structure and 2D interactions of 3B4 with ligand TLF in 0%, 30%, and 50% methanol.

As shown in **Figure** **5**A,B, the average gyration radii of residues within 0.6 nm of the TLF ligand for 3B4 were 1.563, 1.541, and 1.616 nm in 0%, 30%, and 50% methanol, respectively. These findings suggest that the binding cavity of 3B4 was in a more tightly folded state in 30% methanol and a more loosely folded state in 50% methanol. This observation aligns with the changes in the refolding state of 3B4 measured by DSF (Figure 2C).

Additionally, the binding free energy of 3B4 to TLF was found to be −13.51, −15.22, and −8.91 kcal mol^−1^ in 0%, 30%, and 50% methanol, respectively (Figure 5B). These values correspond well with the trends observed in the tolerance curves of 3B4, which initially increased and then sharply decreased (Figure 1B).

Furthermore, interaction analysis revealed that hydrophobic interactions predominated in the binding between 3B4 and the TLF ligand (Figure 5C). Specifically, five residues of 3B4 formed hydrophobic interactions with the ligand TLF in 0% methanol, while six residues and only two residues interacted with the ligand TLF in 30% and 50% methanol, respectively. This suggests that hydrophobic interactions were strengthened in 30% methanol but weakened in 50% methanol.

The results indicate that the binding cavity of 3B4 underwent refolding in 30% methanol, whereas it underwent unfolding in 50% methanol, consistent with the findings from DSC and SLS. It is noteworthy that the comparable binding activities of 3B4 to TLF in 0% and 50% methanol may have resulted from the combinatorial effect of refolding and aggregation, respectively. Therefore, the results of MD simulations support the classification of 3B4 as an antibody type exhibiting “folding favorable and aggregation unfavorable” characteristics. The “folding‐aggregation” hypothesis was well validated through MD simulations for explaining antibody tolerance and provides insights for optimizing methanol concentration in immunoassay systems to achieve the highest sensitivity.

We also observed that Pattern 1 antibodies, represented by 3B3, formed hydrogen bonds with the ligand MAA, while Pattern 2 antibodies, represented by 3B4, did not form such bonds with TLF, suggests a potential relationship between methanol effect patterns and the physicochemical properties of the ligands, particularly their hydrophobicity. To explore this relationship further, we conducted a Pearson correlation analysis between the distribution of ligand‐specific antibodies in Pattern 1 and Pattern 2 antibodies and the physicochemical properties of the ligands (Table S3 and Figure S5, Supporting Information). The results revealed a significant negative correlation between the antibody distribution ratios in Pattern 1 and Pattern 2 and the cLogP values and hydrogen bond donors of the ligands (^**^ *p* = 0.0045 and ^*^ *p* = 0.0466, respectively). This finding suggests that different methanol effect patterns are closely associated with the hydrophobicity and hydrogen bond donors of the ligands. These results provide valuable insights into the factors influencing antibody tolerance in methanol and warrant further investigation to elucidate the underlying mechanisms. Understanding the interplay between antibody properties and ligand characteristics can inform the design and optimization of immunoassay systems for enhanced performance and sensitivity.

### 2.5 The Sequence Basis of Two Antibody Methanol Effect Patterns

The analysis of the constant region and variable region sequences of the 25 antibodies belonging to the two types of methanol effect patterns revealed interesting insights into the sequence basis of antibody tolerance mechanisms in methanol. Firstly, we examined the isotypes of the heavy and light chains for all 25 antibodies and found that both Pattern 1 and Pattern 2 antibodies included the heavy chain isotypes of IgG1 and IgG2a, as well as the light chain isotypes of Kappa and Lambda (Table S4, Supporting Information). This indicates that the tolerance of antibodies in methanol was independent of antibody isotypes in our study.

Next, we conducted multiple sequence alignments of the constant regions of the heavy and light chains of all antibodies. The results showed no significant difference in the constant region sequences between the two patterns of antibodies (Figures S6 and S7, Supporting Information). This suggests that the constant region sequence was independent of the methanol effect patterns, which is consistent with the findings regarding antibody isotype.

These observations indicate that the sequence basis of antibody tolerance in methanol may lie predominantly within the variable regions of the antibodies. Thus, the variable regions of the 25 antibodies were subjected to alignment, and phylogenetic trees were constructed for both the heavy chains and light chains. As shown in Figure S8 (Supporting Information), the two methanol effect patterns could not be clearly distinguished from the phylogenetic trees of either the heavy chains or light chains. However, upon conducting further sequence identity analysis, intriguing results emerged.

It was observed that the identity values within groups for the heavy chains of Pattern 1 and Pattern 2 antibodies were 0.516 and 0.541, respectively. Notably, both of these values were higher than the identity value of 0.497 observed between the two patterns (**Table** **1**; Tables S5 and S6, Supporting Information). This finding suggests that the classification of the two methanol effect patterns may indeed have a sequence basis within the variable regions of the heavy chain.

**Table 1:** The sequence identity analysis within and between two patterns antibodies.

Sequence identity	Within Pattern 1 antibodies	Within Pattern 2 antibodies	Between two patterns antibodies
Heavy chains	0.516	0.541	0.497
Light chains	0.479	0.439	0.475

**Figure 6:**
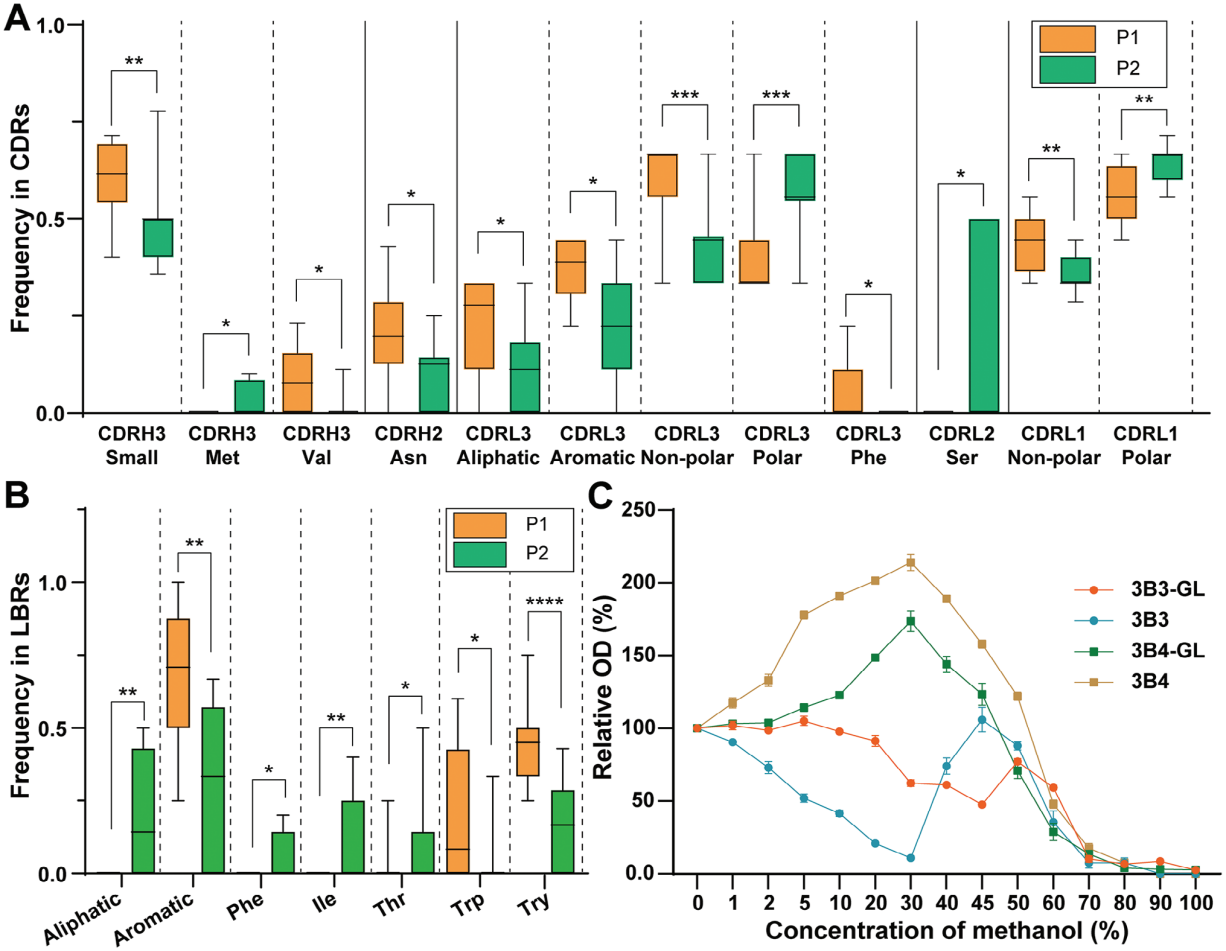
Sequence basis of different methanol effect patterns. A) Residue analysis for CDRs of Pattern 1 and Pattern 2 antibodies. B) Residue analysis for LBRs of Pattern 1 and Pattern 2 antibodies. P1, Pattern 1 antibodies; P2, Pattern 2 antibodies. Asterisks indicate significant differences between the two patterns of antibodies using a two‐tailed Mann‐Whitney test (^*^ *p* <0.05; ^**^ *p* <0.01; ^***^ *p* <0.001; ^****^p <0.0001). C) The tolerance curves of 3B3‐GL and 3B4‐GL in different concentrations of methanol compared with 3B3 and 3B4.

The investigation into the CDRs of the heavy and light chains, crucial components of the antigen‐binding cavity of antibodies, sheds light on the correlation between their characteristics and the two methanol effect patterns observed in antibodies. Analysis of the CDR length revealed no significant differences between the two patterns, as depicted in Figure S9 (Supporting Information). However, a deeper dive into the residue composition of each CDR revealed intriguing insights (Table S7, Supporting Information). Specifically, the analysis unveiled that the small amino acid content, particularly residues like Val, within CDRH3 was significantly higher in Pattern 1 antibodies compared to Pattern 2 antibodies (**Figure** **6**A; Figures S10 and S11, Supporting Information). Given that CDRH3 often plays a pivotal role in antibody function and activity, this disparity implies that the binding cavities of Pattern 1 antibodies may exhibit greater flexibility and susceptibility to compression.

Furthermore, a notable enrichment of aliphatic and aromatic residues was observed in CDRL3 of Pattern 1 antibodies, resulting in a significantly higher non‐polar content compared to Pattern 2 antibodies. Similar trends were found in CDRL1, with Pattern 1 antibodies showing a significantly greater non‐polar content than Pattern 2 antibodies. This suggests that the binding cavities of Pattern 1 antibodies are generally more hydrophobic, whereas those of Pattern 2 antibodies are more hydrophilic. The hydrophobic CDR regions may help maintain or even enhance binding activity when antibodies are in soluble oligomers or aggregated states. In contrast, more hydrophilic CDR regions might experience a marked loss of binding activity under similar conditions.

These structural variations imply that the binding cavity of Pattern 1 antibodies, exemplified by 3B3, with its higher content of small and non‐polar residues, experiences continuous compression in the presence of methanol, particularly at higher concentrations (Figure 4A,B). Conversely, Pattern 2 antibodies, exemplified by 3B4, possess a relatively rigid and hydrophilic binding cavity. While they may experience partial compression in 30% methanol (Figure 2B), the polar nature of methanol enables it to penetrate easily into the binding cavity, leading to unfolding and more severe aggregation in 50% methanol compared to Pattern 1 antibodies (Figure 3B,D). Ultimately, the variability in the residue composition of these CDRs shapes the distinct properties of antigen‐binding cavities, thereby influencing the activities of antibodies in methanol and delineating the two observed methanol effect patterns.

The differences in ligand‐binding residues (LBRs) within the CDR regions are likely to play a crucial role in determining the methanol effect patterns of antibodies. To explore these LBRs, we modeled the other 23 antibodies alongside 3B3 and 3B4 and docked their respective ligands. Subsequently, the 2D interactions between antibodies and their ligands were illustrated (Figures S12 and S13, Supporting Information), and the LBRs of antibodies were listed (**Table** **2**). Notably, both Pattern 1 and Pattern 2 antibodies exhibited over 50% non‐polar content in their LBRs (Figure S14, Supporting Information), suggesting that hydrophobic forces may dominate the interactions between antibodies and ligands in both cases.

**Table 2:** The ligand‐binding residues (LBRs) of 25 antibodies to NSAIDs.

Patterns	Antibodies	LBRs
Pattern 1	1G8	**Tyr33(A)**, **Arg98(A)**, **Pro100(A)**, **Ser32(B)**, Tyr33(B), **Tyr37(B)**
Pattern 1	6A8	Trp54(A), **Asn58(A)**, **Tyr60(A)**, **Trp106(A)**, **Tyr34(B)**, **Trp93(B)**
Pattern 1	1F2	**Trp93(B)**, **Trp54(A)**, **Trp106(A)**, **Tyr34(B)**, **Tyr98(B)**
Pattern 1	2B10	Tyr60(A), **Trp106(A)**, **Tyr34(B)**, **Trp93(B)**, **Tyr98(B)**
Pattern 1	11F5	**Tyr60(A)**, Ser95(B), **Trp54(A)**, **Trp106(A)**, **Tyr34(B)**, **Trp93(B)**
Pattern 1	3B3	**Tyr38(A)**, Tyr110(B), Ser36(A), Trp107(A)
Pattern 1	20A2	**Trp54(A)**, **Asp56(A)**, Asn58(A), **Tyr60(A)**, Tyr106(A), Tyr34(B), **Trp93(B)**, **Tyr98(B)**
Pattern 1	2B11M	Thr33(A), **Asn52(A)**, Gly50(A), **Gly57(A)**, **Thr58(A)**, Asn59(A), **Tyr100(A)**, **Tyr101(A)**
Pattern 1	10C2	**Arg46(B)**, **Tyr91(B)**, **Ser99(A)**, **Pro100(A)**, Asn32(B), **Tyr49(B)**
Pattern 1	2H5	**His35(A)**, **Ser99(A)**, **Asp100(A)**, **Tyr101(A)**, **Tyr49(B)**, Asp50(B)
Pattern 1	3C10	**Ser31(A)**, **Asp33(A)**, **Tyr101(A)**, **Tyr37(B)**
Pattern 1	5G2	**Arg99(A)**, **Asp100(A)**, **Trp101(A)**, **Tyr32(B)**, Tyr91(B), **Tyr92(B)**
Pattern 1	2A2	**Arg101(A)**, **Arg101(A)**, **Tyr92(B)**, **Tyr34(A)**, **His91(B)**
Pattern 1	1E6	**Tyr99(B)**, **Tyr33(A)**, **Tyr37(B)**, **His96(B)**
Pattern 2	2D1	**Arg33(A)**, **Arg33(A)**, **His91(B)**, **Ser98(A)**, Thr99(A), Tyr32(B), **Tyr50(B)**, **Gly90(B)**
Pattern 2	4H11	**Trp54(A)**, **Trp55(A)**, **Asn56(A)**, Glu58(A), His102(A), **Tyr108(A)**
Pattern 2	1F5	Arg50(A), Ile52(A), Ala57(A), **Ile101(A)**, **Pro102(A)**, **Tyr34(B)**
Pattern 2	6D7	Leu59(A), Arg31(B)
Pattern 2	1A7	**Tyr96(B)**, **Tyr96(B)**, **Lys99(A)**, **Gly101(A)**, **Phe102(A)**, **Tyr91(B)**, Ile92(B)
Pattern 2	2C10	**Ser50(B)**, **Gly101(B)**, **Phe102(A)**, **Thr31(B)**, **Asn32(B)**, **Tyr91(B)**
Pattern 2	7G8	**Tyr91(B)**, **Lys99(A)**, **Gly101(A)**, **Phe102(A)**, **Ser50(B)**
Pattern 2	8B1	**Tyr33(A)**, **Ala50(A)**, Asp57(A), **Thr58(A)**, **Tyr59(A)**, Ile102(A), **Leu98(B)**
Pattern 2	3B4	Ser27(A),Ile32(A), Ile100(A), Pro113(A), Met115(A)
Pattern 2	4A5	**Tyr34(A)**, Arg53(A), Phe51(A), **Asp59(A)**, **His91(B)**, **Tyr92(B)**, **Thr94(B)**
Pattern 2	2B11T	Ile57(A), Thr58(A), Asp59(A), **Thr94(B)**

(A), heavy chain; (B), light chain. The germline‐encoded residues are boldly shown in the Table.

However, significant differences were observed in the types of residues present in the LBRs of Pattern 1 and Pattern 2 antibodies. Specifically, Pattern 1 antibodies exhibit significantly higher levels of aromatic residues, particularly Trp and Tyr, whereas Pattern 2 antibodies had significantly higher levels of aliphatic residues, especially Ile (Figure 6B). In addition to providing hydrophobic force, the Trp and Tyr residues in Pattern 1 antibodies could also form H‐bond interactions (Figure S12, Supporting Information), while the aliphatic residues lack H‐bond/charge‐charge interaction capabilities.^[^ ^36^ ^]^ These substantial differences in residue types within the LBRs of antibodies belonging to the two methanol effect patterns may underlie their unique tolerance to methanol, although the precise mechanism remains to be elucidated.

Further analysis of the LBRs for the 25 antibodies revealed that 72.3% of them were germline‐encoded (bold shown in Table 2). This finding suggests that the two methanol effect patterns of antibodies may be delineated by their germline antibodies (GL), which represent the initial stage of antibody evolution in vivo.^[^ ^7^ ^]^ Consequently, we inferred the germline antibodies of 3B3 and 3B4, denoted as 3B3‐GL and 3B4‐GL, respectively, and expressed them using the HEK 293 cell expression system (Figure S15, Supporting Information).

To validate the binding activity of 3B3‐GL and 3B4‐GL, we constructed antibody dilution curves and competitive inhibition curves using their corresponding coating antigens and ligands. As shown in Figure S16A (Supporting Information), the OD values of the antibody dilution curves increased significantly with the concentration of germline antibodies, indicating that 3B3‐GL and 3B4‐GL effectively bound their coating antigens. Similarly, in Figure S16B (Supporting Information), both 3B3‐GL and 3B4‐GL exhibited specific binding to their ligands, with IC_50_ values of 1218.7 and 33.3 ng mL^−1^, respectively, as determined by icELISA. These results confirm the successful inference and preparation of the germline antibodies of 3B3 and 3B4.

Subsequently, we characterized the binding activity of 3B3‐GL and 3B4‐GL in methanol using ncELISA to evaluate their methanol effect patterns. As shown in Figure 6C, 3B3‐GL exhibited a similar “Valley‐Peak” pattern curve consistent with 3B3, while 3B4‐GL displayed a typical “Peak” pattern curve consistent with 3B4. These findings suggest that the antibodies retained certain methanol effect patterns throughout the process of somatic hypermutation and affinity maturation in vivo. In essence, germline antibodies appear to play a crucial role in defining the methanol effect patterns of mature antibodies, indicating that germline antibodies with high tolerance may serve as valuable materials and provide essential insights for the development of tolerant antibodies in organic solvents in vitro. Overall, we believe that the tolerance characteristics of germline antibodies have the potential to guide the discovery and engineering of methanol‐tolerant antibodies based on these findings.

## 3 Conclusion

In this work, we identified two distinct methanol effect patterns of antibodies for analytical purposes by employing the ncELISA approach on 25 antibodies. These patterns were classified as the “Valley‐Peak” pattern and the “Peak” pattern. We determined that these patterns were not influenced by changes in the secondary structures of antibodies but were instead correlated with the folding and aggregation behaviors of antibodies. Therefore, we proposed the “folding‐aggregation” hypothesis for the first time, which was subsequently validated by MD simulations. Furthermore, our analysis of the sequence basis of antibody methanol effect patterns revealed significant differences in residue types within the CDRs and LBRs between the two patterns. Notably, the germline antibodies were found to define the methanol effect patterns of mature antibodies, suggesting their crucial role in antibody tolerance. Overall, this study provides insights into the structural and sequence basis of different methanol effect patterns of antibodies, offering potential applications in the discovery and engineering of tolerant antibodies and the development of robust immunoassays in organic solvents.

## 4 Experimental Section

### Reagents and Apparatus

Analginum, carprofen (CPF), flunixin (FLU), meloxicam (MLX), 4‐methylaminoantipyrine (MAA,), and tolfenamic acid (TLF) were sourced from J&K Scientific (Beijing, China). Methanol was acquired from Thermo Fisher Scientific (CA, USA). A protein A column was purchased from Smart‐Life Science (Beijing, China). Goat anti‐mouse IgG‐peroxidase conjugate was obtained from Jackson ImmunoResearch Laboratories, Inc. (West Grove, PA, USA). Phosphate‐buffered saline (PBS, 10 mm, pH 7.2) served as the working buffer. Different methanol concentrations were prepared in PBS buffer. The 25 tested monoclonal antibodies were prepared by the group following a standard protocol^[^ ^37^ ^]^ and will be described elsewhere in detail.

### The Measurement of Antibody Tolerance in Methanol

To investigate antibody tolerance in organic solvents, 25 antibodies targeting NSAIDs were utilized, comprising five for CPF (3C10, 5G2, 1A7, 2C10, and 7G8), two for FLU (2H5 and 6D7), nine for MAA (1G8, 6A8, 1F2, 2B10, 11F5, 3B3, 20A2, 2D1, and 4H11), the metabolite of analginum, three for MLX (2B11M, 10C2, and 1F5), and six for TLF (2A2, 1E6, 8B1, 3B4, 4A5, and 2B11T). The structures of CPF, FLU, MAA, MLX, and TLF are depicted in Figure S1 (Supporting Information). Antibody tolerance in various concentrations of methanol was assessed using a noncompetitive enzyme‐linked immunosorbent assay (ncELISA) protocol, as outlined below: Polystyrene ninety‐six well microtiter plates were coated with coating antigen in coating buffer (100 µL well^−1^) and incubated for 2 h at 37 °C. Following three washes with washing buffer, the plates were blocked with blocking buffer (300 µL/well) for 1 h at 37 °C. Antibody samples (100 µL well^−1^), prepared in different concentrations of methanol ranging from 0% to 100%, were added to the wells. After incubation for 30 min at 37°C, unbound compounds were removed by washing with washing buffer. Goat anti‐mouse IgG‐HRP (diluted 1:5000 in dilution buffer, 100 µL well^−1^) was added, followed by incubation for 30 min at 37°C and subsequent washing three times. Subsequently, the 3,3′,5,5′‐Tetramethylbenzidine (TMB) substrate solution (100 µL well^−1^) was added and incubated for 15 min at room temperature (RT) before the addition of stopping reagent (50 µL well^−1^). The buffer solutions used in the experiment are listed in the supporting information. The working concentrations of antibodies and coating antigen are listed in Table S1 (Supporting Information). Optical density (OD) values were measured at 450 nm. The relative OD value was calculated using the following formula:

(1) $$\begin{array}{ccc} & & \mathrm{Relative}\;\mathrm{OD}\;\mathrm{value}\\ & & \;=(\mathrm{OD}\;\mathrm{value}\;\mathrm{in}\;\mathrm{buffer}\;\mathrm{with}\;\mathrm{different}\;\mathrm{concentrations}\;\mathrm{of}\;\mathrm{methanol}/\\ & & \;\;\;\;\mathrm{OD}\;\mathrm{value}\;\mathrm{in}\;\mathrm{buffer}\;\mathrm{without}\;\mathrm{methanol})\times 100\% \end{array}$$

### The Determination of the Secondary Structure of Antibodies in Methanol

Circular dichroism (CD) spectra of antibodies in varying concentrations of methanol were acquired using a Chirascan Plus spectrometer (Applied Photophysics Ltd., UK) in the far region. Samples were contained within a 0.1 cm pathlength quartz cell, and measurements were conducted at RT. The absorbance wavelength was set between 180 and 260 nm, with fixed scanning intervals and scanning speed of 1.0 and 1.0 nm ^−1^s, respectively. Antibody concentration was maintained at 0.2 mg mL^−1^ in different methanol concentrations, with each methanol concentration serving as a control and subtracted from the actual sample data. The obtained data were presented as milli‐degree (mdeg) values, with each spectrum being the result of three accumulations. The secondary structure analysis of antibodies, including α‐helix, β‐sheet, β‐turn, and random coil, was performed using CDNN 2.1 software.

### The Determination of Antibody Folding and Aggregation in Methanol

The folding behavior of antibodies at different methanol concentrations was determined using differential scanning fluorimetry (DSF) with an Uncle multifunctional protein stability analysis system (Unchained Labs, USA). The intrinsic fluorescence intensity of tryptophan was monitored during the process. Nine microliters of antibody sample (5 mg/mL) with varying methanol concentrations were loaded into the Uni sample loader in triplicate, with temperatures increasing from 25 to 42 °C at a rate of 1.3 °C min^−1^. The barycentric mean (BCM) of the maximum emission wavelength of fluorescence was calculated using UNcle Analysis software (Version 4.0).

The aggregation behavior of antibodies at different methanol concentrations was evaluated through static light scattering (SLS) using the UNcle system. Purified protein samples (5 mg/mL) were heated from 25 to 42 °C at a rate of 1.3 °C min^−1^. The intensity of the scattered light at 266 nm was measured and calculated using UNcle Analysis software (Version 4.0).

### Antibody Modeling, Molecular Docking, and Molecular Dynamics Simulation

The variable regions of antibodies 3B3 and 3B4 were homology modeled using Discovery Studio 2019 (DS) software. Molecular docking of antibodies and ligands was conducted utilizing the CDOCKER module within DS. Following the docking process, wherein ligands were placed into the top‐ranked cavity of the antibody, the binding pose with the highest score for each antibody‐ligand complex was selected. The top‐scoring binding poses were then subjected to all‐atomic, explicit water molecular dynamics (MD) simulations employing the GROMACS simulation package.^[^ ^38^ ^]^ Antibodies and ligands were parameterized with AMBER 14SB^[^ ^39^ ^]^ and AMBER GAFF force fields^[^ ^40^ ^]^ respectively. The simulation system was solvated in a rectangular box utilizing the TIP3P water model and neutralized by adding counterions (Na^+^/Cl^−^). The distance between the water box edge and the solute surface was maintained at 1.2 nm. Long‐range electrostatic interactions were managed using the particle grid method, with a van der Waals interaction cut‐off radius set at 1.2 nm. Subsequently, the system was energy‐minimized using the steepest descent and conjugate gradient methods, each for 1000 steps of optimization, followed by a 2 ns position‐restricted MD simulation under NPT ensembles. Finally, each system underwent a 100 ns simulation with all constraints removed, maintaining a temperature of 310 K (V‐rescale thermostat), atmospheric pressure (Parrinello‐Rahman barostat), and periodic boundary conditions utilizing the Verlet cut‐off scheme. The binding free energy of the antibody‐ligand complex was computed using the gmx_MMPBSA toolkit based on mmpsa.py. The structure of the antibody‐ligand complex was visualized using VMD software, and the two‐dimensional (2D) interaction diagram of the antibody‐ligand complex was generated using Ligplot software.^[^ ^41^ ^]^

### Germline Antibody Analysis, Expression, and Validation

The germline antibodies of 3B3 and 3B4, denoted as 3B3‐GL and 3B4 ‐GL, were inferred using the IMGT/V‐QUEST tools of available in the IMGT database.^[^ ^42^ ^]^ Subsequently, 3B3‐GL and 3B4 ‐GL were synthesized via transient transfection of HEK 293 cells, following a previously described protocol with minor adjustments.^[^ ^43^ ^]^ Briefly, cells were suspended in an expression medium, and the recombinant plasmids containing heavy and light chains were introduced, followed by immediate addition of linear PEI MAX (Polysciences Inc., Warrington, PA, USA) for 3 h at 37 °C. Subsequently, the cell density was reduced in ExCell VPRO medium. After 7 days of culturing, the supernatants were filtered and diluted with PBS (pH 7.2) at a 1:1 ratio, then loaded onto a Protein A column using the ÄKTA pure protein purification system. Antibodies were eluted from the column using 0.1 m citrate (pH 3.0) in 1.0 m Tris‐HCl (pH 9.0). The purified IgG samples were buffer‐exchanged via dialysis. The identification of germline antibodies was confirmed through reduced and non‐reduced sodium dodecyl sulphate‐polyacrylamide gel electrophoresis (SDS‐PAGE). Additionally, the antigen‐recognition properties of germline antibodies were validated through antibody dilution assay and indirect competitive enzyme‐linked immunosorbent assay (icELISA) (See in supporting information). The binding activity of germline antibodies at different methanol concentrations was assessed using ncELISA.

### Statistical Analysis

Statistical analysis was performed using GraphPad Prism 8.0 (GraphPad Software, Inc.). A two‐tailed Mann‐Whitney test was used for all statistical analyses. (^*^ *p* <0.05; ^**^ *p* <0.01; ^***^ *p* <0.001; ^****^ *p* <0.0001).

## Conflict of Interest

The authors declare no conflict of interest.

## Supporting information

Supporting Information

## Data Availability

The data that support the findings of this study are available from the corresponding author upon reasonable request.
