# Supplementary material for: Investigation of Antibody Tolerance in Methanol for Analytical Purposes: Methanol Effect Patterns and Molecular Mechanisms
Source: Adv Sci (Weinh). 2024 Jun 18;11(31):2402050. doi: 10.1002/advs.202402050 (PMC11336977; doi:10.1002/advs.202402050)
Supplement: Supplementary file 1 — Supporting Information [file ADVS-11-2402050-s001.docx]

**Supplementary information**

for

**Investigation of Antibody Tolerance in Methanol for Analytical Purposes:** **Methanol Effect Patterns and Molecular Mechanisms**

Yingjie Zhang^#^, Jiafei Mi^#^, Weilin Wu, Jie Fei, Bochen Lv, Xuezhi Yu, Kai Wen, Jianzhong Shen* and Zhanhui Wang*

*National Key Laboratory of Veterinary Public Health and Safety, Beijing Key Laboratory of Detection Technology for Animal-Derived Food, College of Veterinary Medicine, China Agricultural University, 100193 Beijing, People’s Republic of China*

^#^*Yingjie Zhang and Jiafei Mi* contributed equally to this work

**Corresponding authors: Jianzhong Shen* and *Zhanhui Wang*

*Tel: +86-10-6273 4565*

*Fax: +86-10-6273 1032*

*E-mail: sjz@cau.edu.cn (Jianzhong Shen); wangzhanhui@cau.edu.cn (Zhanhui Wang)*

# Table of Contents for Supporting Information

|  | Contents | Pages |
| --- | --- | --- |
| 1 | Buffers | S3 |
| 2 | ELISA methodology validation and development | S3-S6 |
| 3 | Antibody sequencing | S6 |
| 4 | Supporting Figures and Tables | S7-S31 |
| 5 | References | S32-S33 |

**1. Buffers**

The common buffer solutions used in the experiment are listed:

(1) Coating buffer (0.05 mol/L, carbonate buffer, pH 9.6);

(2) Blocking buffer (10% skim milk powder (w/v) dissolved in PBS);

(3) Washing buffer (PBST, 0.01 mol/L PBS buffer with 0.05% Tween-20 (v/v), pH 7.4);

(4) Phosphate buffer solution (PBS buffer, 0.01 mol/L, pH 7.4);

| NaH_2_PO_4_·2H_2_O | 0.59 g |
| --- | --- |
| Na_2_HPO_4_·12H_2_O | 2.9 g |
| NaCl | 8.5 g |
| KCl | 0.2 g |
| Ultrapure water | Volume to 1 L |

(5) Antibody dilution buffer (PBS buffer with 0.2% albumin, (w/v));

(6) Goat anti-mouse IgG (HRP labeled) dilution buffer (PBS buffer containing 5% albumin (w/v));

(7) 3,3′,5,5′-Tetramethylbenzidine (TMB) Two-Component Substrate solution (TMB/H_2_O_2_)

(8) Stopping reagent (2 M H_2_SO_4_).

**2. ELISA methodology validation and development**

The effect of methanol on the coating and blocking system of the noncompetitive enzyme-linked immunosorbent assay (ncELISA) was assessed by treating microtiter plates with varying concentrations of methanol (0%, 20%, 40%, 60%, 80%, and 100% v/v) after the coating and blocking steps. Following a 30-minute incubation at 37°C, unbound compounds were removed by washing with buffer. Antibody samples (50 µL/well) and PBS (50 µL/well) were prepared and added to the wells. After a 30-minute incubation period at 37℃, unbound compounds were removed by washing the plates with washing buffer. Subsequently, goat anti-mouse IgG-HRP (diluted at 1:5000 in dilution buffer) was added to each well (100 µL/well) and incubated for an additional 30 minutes at 37°C, followed by three washes. The substrate solution (100 µL/well) was then added and incubated for 15 minutes at room temperature before the addition of the stopping reagent (50 µL/well). Optical density (OD) values were measured at 450 nm to evaluate any interference caused by methanol on the ncELISA system.

To assess the effect of methanol on the non-specific adsorption of the second antibody to the coated plates, the ncELISA were constructed with a few modifications. After the coating and blocking steps, the microtiter plates were treated with varying concentrations of methanol (0%, 20%, 40%, 60%, 80%, and 100% v/v). Following a 30-minute incubation at 37°C, unbound compounds were removed by washing with buffer. Subsequently, the plates were treated with goat anti-mouse IgG-HRP (diluted at 1:5000 in dilution buffer, 100 µL/well) and incubated for an additional 30 minutes at 37°C. After incubation, unbound compounds were removed by washing with a washing solution, and the plates were developed by adding substrate solution (100 µL/well) and incubating for 15 minutes at room temperature (RT). Finally, the reaction was stopped by adding stopping reagent (50 µL/well), and OD values were measured at 450 nm to evaluate any potential impact of methanol on non-specific adsorption.

To assess the effect of methanol on the non-specific adsorption of the primary antibodies and second antibody to unlabeled OVA, the ncELISA were constructed with a few modifications. Polystyrene ninety-six well microtiter plates were coated with OVA instead of the coating antigen in coating buffer (100 µL/well) and incubated for 2 h at 37℃. Following three washes with washing buffer, the plates were blocked with blocking buffer (300 μL/well) for 1 h at 37℃. Antibody samples (100 µL/well), prepared in different concentrations of methanol ranging from 0% to 100%, were added to the wells. After incubation for 30 min at 37℃, unbound compounds were removed by washing with washing buffer. Goat anti-mouse IgG-HRP (diluted 1:5000 in dilution buffer, 100 µL/well) was added, followed by incubation for 30 min at 37℃ and subsequent washing three times. Subsequently, the 3,3′,5,5′-Tetramethylbenzidine (TMB) substrate solution (100 µL/well) was added and incubated for 15 min at room temperature (RT) before the addition of stopping reagent (50 µL/well).

The affinity of the antibodies was assessed using an indirect competitive enzyme-linked immunosorbent assay (icELISA) protocol as follows: Polystyrene ninety-six well microtiter plates were coated with the coating antigen in coating buffer (100 µL/well) and incubated for 2 hours at 37℃. Following three washes with washing buffer, the plates were blocked with blocking buffer (300 μL/well) for 1 hour at 37℃. Antibody samples (50 µL/well) and ligand (50 µL/well) were prepared and added to the wells. After a 30-minute incubation period at 37℃, unbound compounds were removed by washing the plates with washing buffer. Subsequently, goat anti-mouse IgG-HRP (diluted at 1:5000 in dilution buffer, 100 µL/well) was added and incubated for 30 minutes at 37℃, followed by three additional washes. The substrate solution (100 µL/well) was added and allowed to incubate for 15 minutes at RT before the addition of stopping reagent (50 µL/well). Finally, the assay was terminated, and the absorbance was measured at 450 nm to determine the antibody-antigen affinity.

**3. Antibody sequencing**

The total RNA was extracted from hybridoma cells using the Rneasy Mini Kit. Subsequently, the RNA was promptly utilized for cDNA synthesis, followed by the acquisition of target sequences through RT-PCR. The PCR products were sent to Genwiz (Suzhou, China) for sequencing. The resulting gene sequences were submitted to the Expasy database (https://web.expasy.org) to obtain the variable and constant amino acid sequences of 25 NSAIDs antibodies.

# 4. Supporting Figures and Tables

|  | Supporting Figures and Tables | Pages |
| --- | --- | --- |
| **Fig. S1** | The chemical structures of carprofen (CPF), flunixin (FLU), 4-methylaminoantipyrine (MAA, the metabolite of analginum (ANG)), meloxicam (MLX) and tolfenamic acid (TLF). | S9 |
| **Fig. S2** | Validation of ncELISA methodology for the measurement of antibody tolerance in methanol. | S10 |
| **Fig. S3** | Antibody modeling and model validation. | S11 |
| **Fig. S4** | The complex structures of mAb 3B3-MAA (A) and mAb 3B4-TLF (B). | S12 |
| **Fig. S5** | Investigation the relationship between the physicochemical properties of ligand and the distribution ratio of P1/P2 patterns. | S13 |
| **Fig. S6** | The multiple sequence alignment of antibody heavy chain constant regions. | S14 |
| **Fig. S7** | The multiple sequence alignment of antibody light chain constant regions. | S15 |
| **Fig. S8** | The phylogenetic trees of the variable region of heavy chains (A) and light chains (B) for two patterns antibodies. | S16 |
| **Fig. S9** | The CDR length analysis of pattern 1 and pattern 2 antibodies. | S17 |
| **Fig. S10** | The residue analysis of CDRH3 (A), CDRH2 (B) and CDRH1 (C) of pattern 1 and pattern 2 antibodies. | S18 |
| **Fig. S11** | The residue analysis of CDRL3 (A), CDRL2 (B) and CDRL1 (C) of pattern 1 and pattern 2 antibodies. | S19 |
| **Fig. S12** | The 2D interaction of Pattern 1 antibodies with their ligands. | S20 |
| **Fig. S13** | The 2D interaction of Pattern 2 antibodies with their ligands. | S21 |
| **Fig. S14** | The residue analysis of ligand-binding residues (LBRs) of pattern 1 and pattern 2 antibodies. | S22 |
| **Fig. S15** | The non-reducing and reducing polyacrylamide gel electrophoresis (PAGE) of 3B3-GL (A) and 3B4-GL (B). | S23 |
| **Fig. S16** | The antigen-recognition properties of 3B3-GL and 3B4-GL were verified by antibody dilution curves (A) and competitive inhibition curves (B). | S24 |
| **Table S1** | The working concentrations of antibodies and coating antigen used in this work. | S25 |
| **Table S2** | The methanol effect of reported antibodies to chemical compounds in methanol. | S26 |
| **Table S3** | The distribution of ligand-specific antibodies in Pattern 1 and Pattern 2 antibodies and the physicochemical properties of ligand. | S27 |
| **Table S4** | The heavy chain and light chain isotypes of 25 antibodies to NSAIDs. | S28 |
| **Table S5** | The sequence identity analysis of heavy chains of 25 NSAIDs antibodies. | S29 |
| **Table S6** | The sequence identity analysis of light chains of 25 NSAIDs antibodies. | S30 |
| **Table S7** | The categories of residues used in this study. | S31 |

**Fig. S1.** The chemical structures of carprofen (CPF), flunixin (FLU), 4-methylaminoantipyrine (MAA, the metabolite of analginum (ANG)), meloxicam (MLX) and tolfenamic acid (TLF). In this work, 25 model antibodies against those five chemical drugs: five for CPF, two for FLU, nine for MAA, three for MLX, and six for TLF.


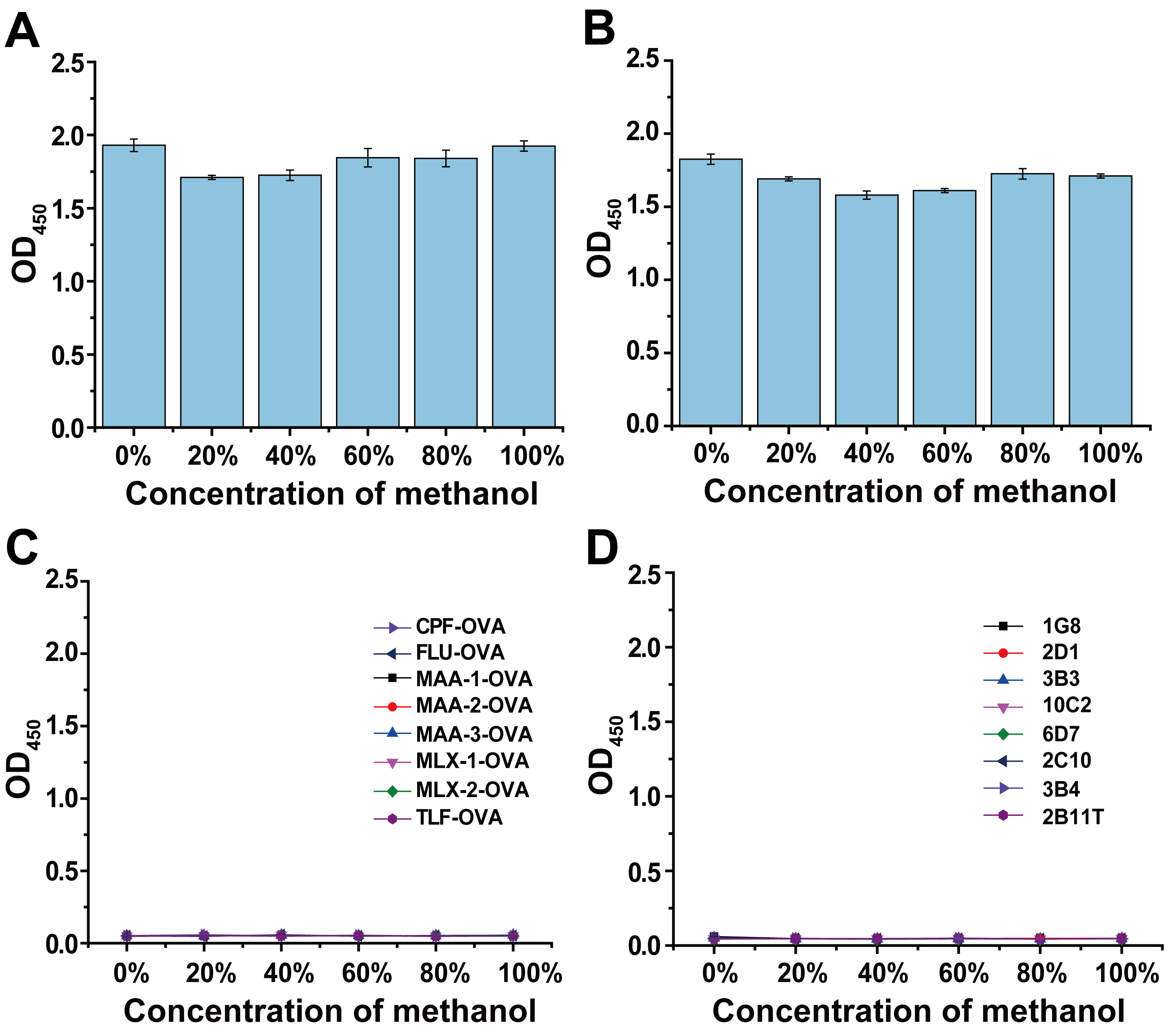


**Fig. S2.** Validation of ncELISA methodology for the measurement of antibody tolerance in methanol. The effect of methanol on the coating and blocking system was tested by two mAb/coating antigen pairs, mAb 1G8/MAA-1-OVA (A) and mAb 10C2/MLX-1-OVA (B). The effect of methanol on the non-specific adsorption of second antibody to coating antigens (CPF-OVA, FLU-OVA, MAA-1-OVA, MAA-2-OVA, MAA-3-OVA, MLX-1-OVA, MLX-2-OVA and TLF-OVA) was tested (C). The effect of methanol on the non-specific adsorption of primary antibodies (1G8, 2D1, 3B3, 10C2, 6D7, 2C10, 3B4 and 2B11T) and second antibody to OVA was tested (D). Those coating antigens and primary antibodies are prepared according to standard procedure by our group and will be described elsewhere. OVA, ovalbumin.


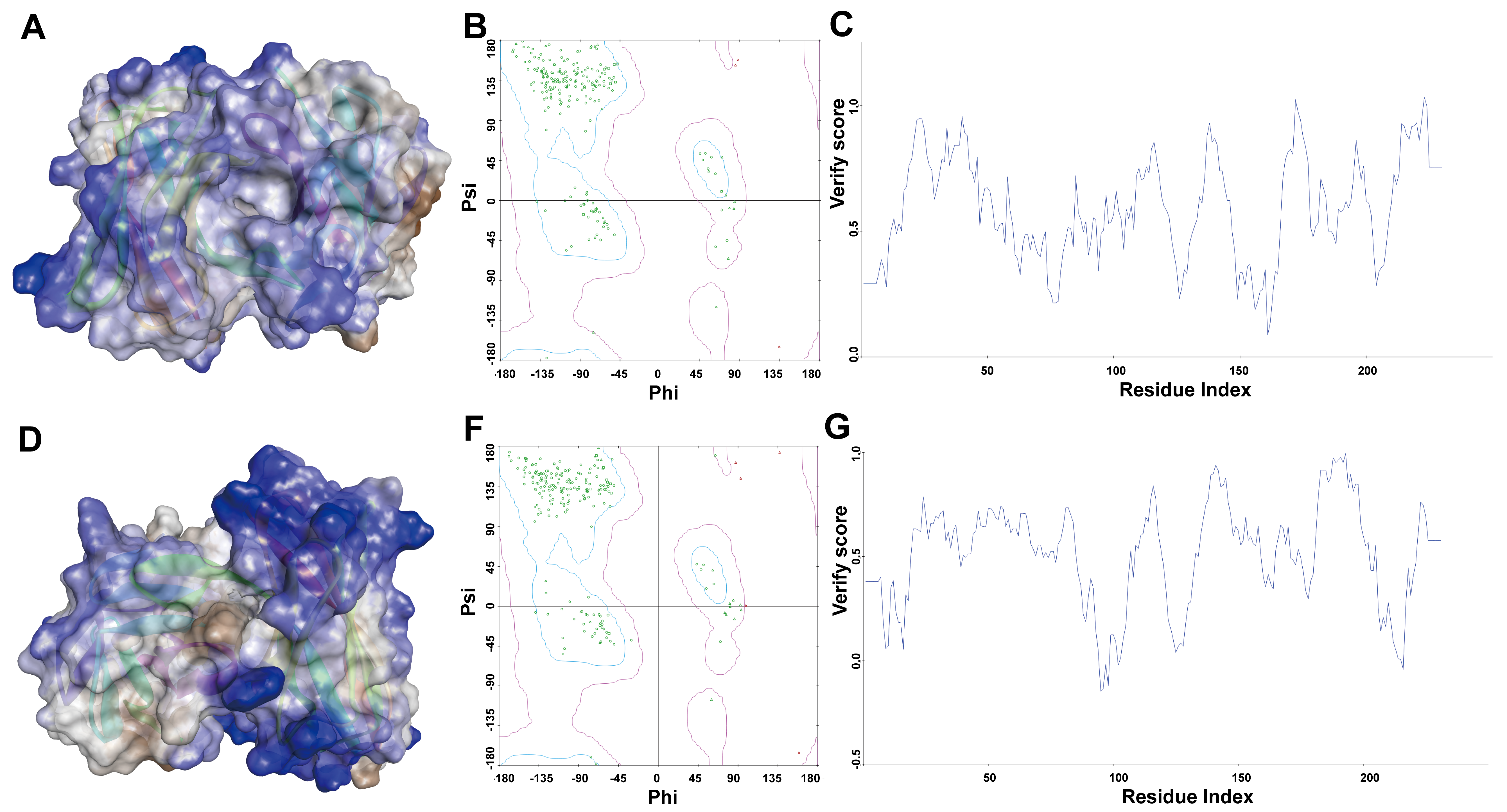


**Fig. S3.** Antibody modeling and model validation. The variable regions of antibodies 3B3 (A) and 3B4 (D) were modeled by Discovery Studio 2019 (DS) software.

The best models were validated by Ramachandran plots and Profile-3D plots of mAb 3B3 (B–C) and mAb 3B4 (F–G).


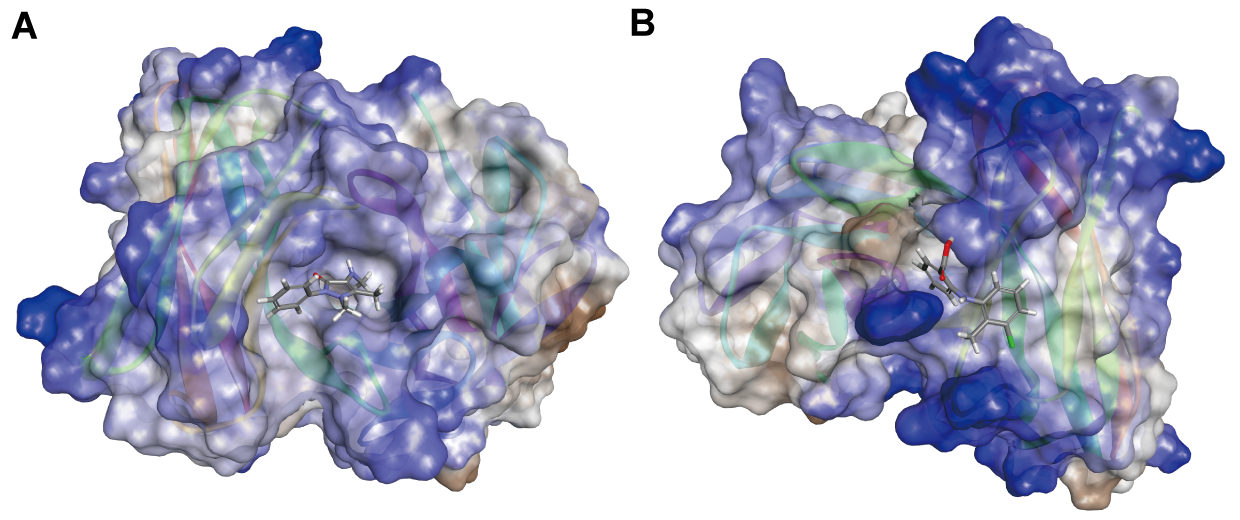


**Fig. S4.** The complex structures of the variable regions of antibodies 3B3 and MAA (A), and the variable regions of antibodies 3B4 and TLF (B). The molecular docking of antibodies and ligands is carried out by the CDOCKER module of DS software.


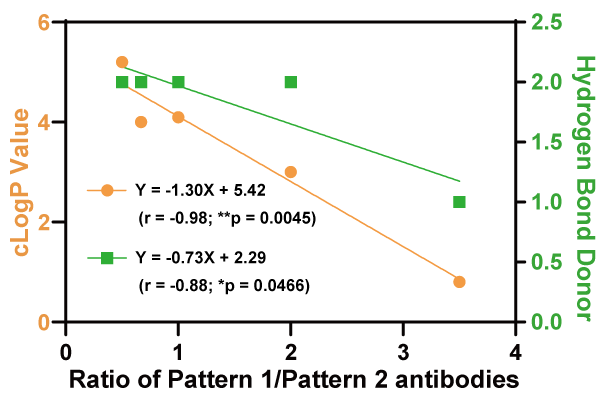


**Fig. S5.** Investigation the relationship between the physicochemical properties of ligand and the distribution ratio of P1/P2 patterns. Pearson correlation coefficient (r) and two-tailed p values (p) were shown.

**
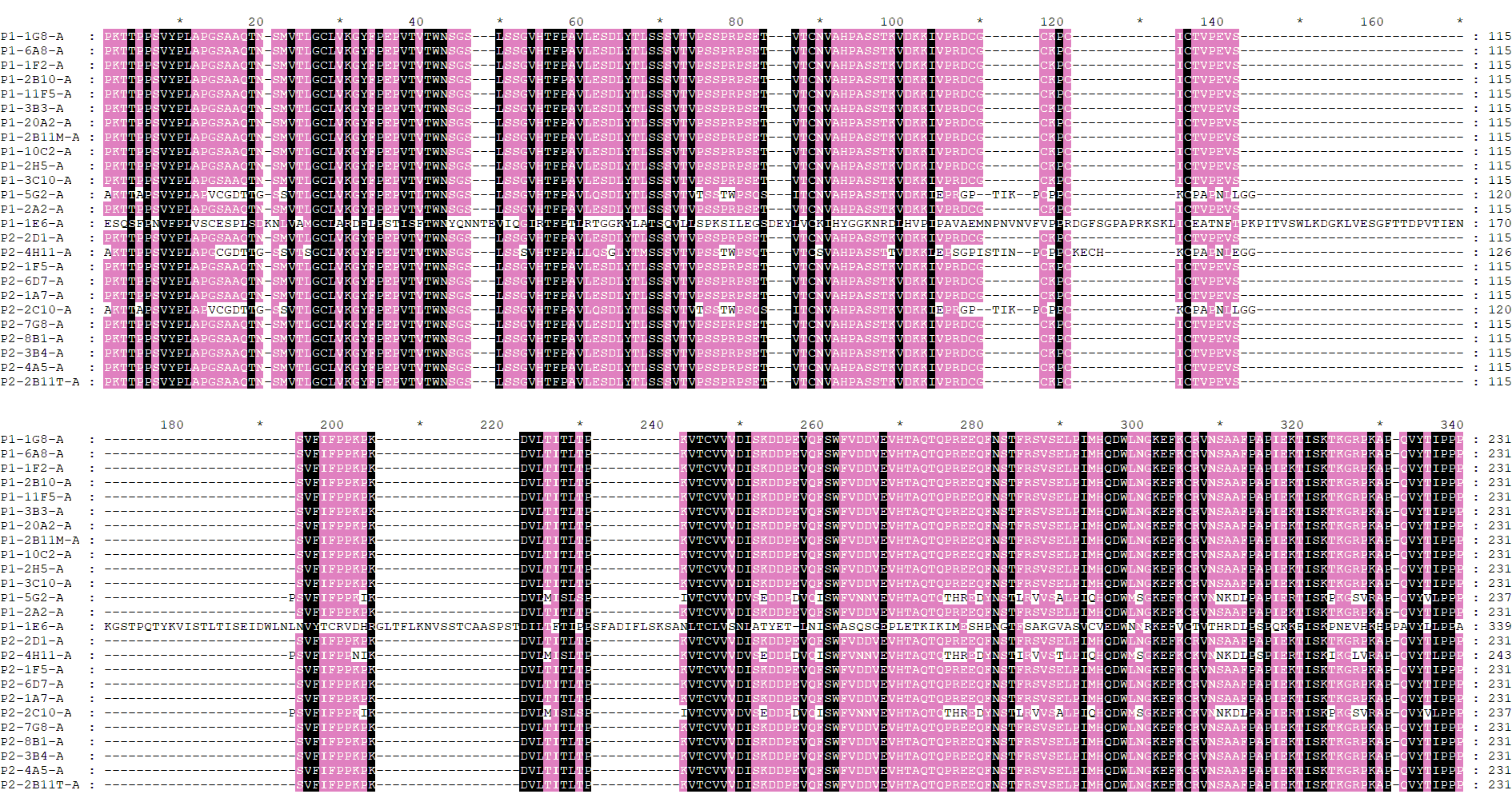
**

**Fig. S6.** The multiple sequence alignment of antibody heavy chain constant regions. The multiple sequence alignment was conducted by ClustalW method using the MEGA and GeneDoc software. Consistent residues are shown on a black background and similar residues are shown on a magenta background. P1, Pattern 1 antibodies; P2, Pattern 2 antibodies; A, the constant region of heavy chain.

**
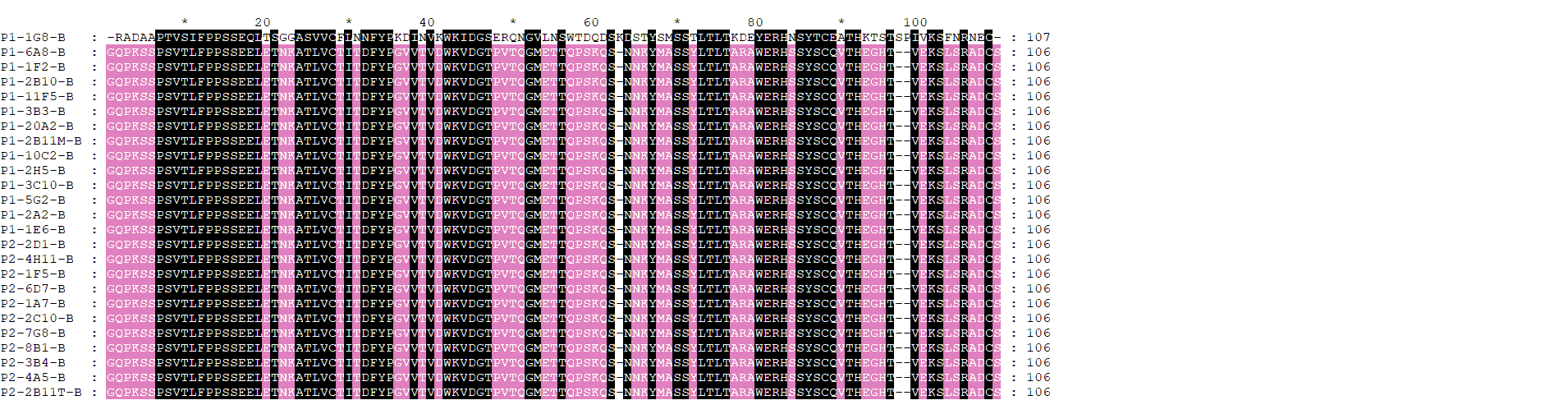
**

**Fig. S7.** The multiple sequence alignment of antibody light chain constant regions. The multiple sequence alignment was conducted by ClustalW method using the MEGA and GeneDoc software. Consistent residues are shown on a black background and similar residues are shown on a magenta background. P1, Pattern 1 antibodies; P2, Pattern 2 antibodies; B, the constant region of light chain.


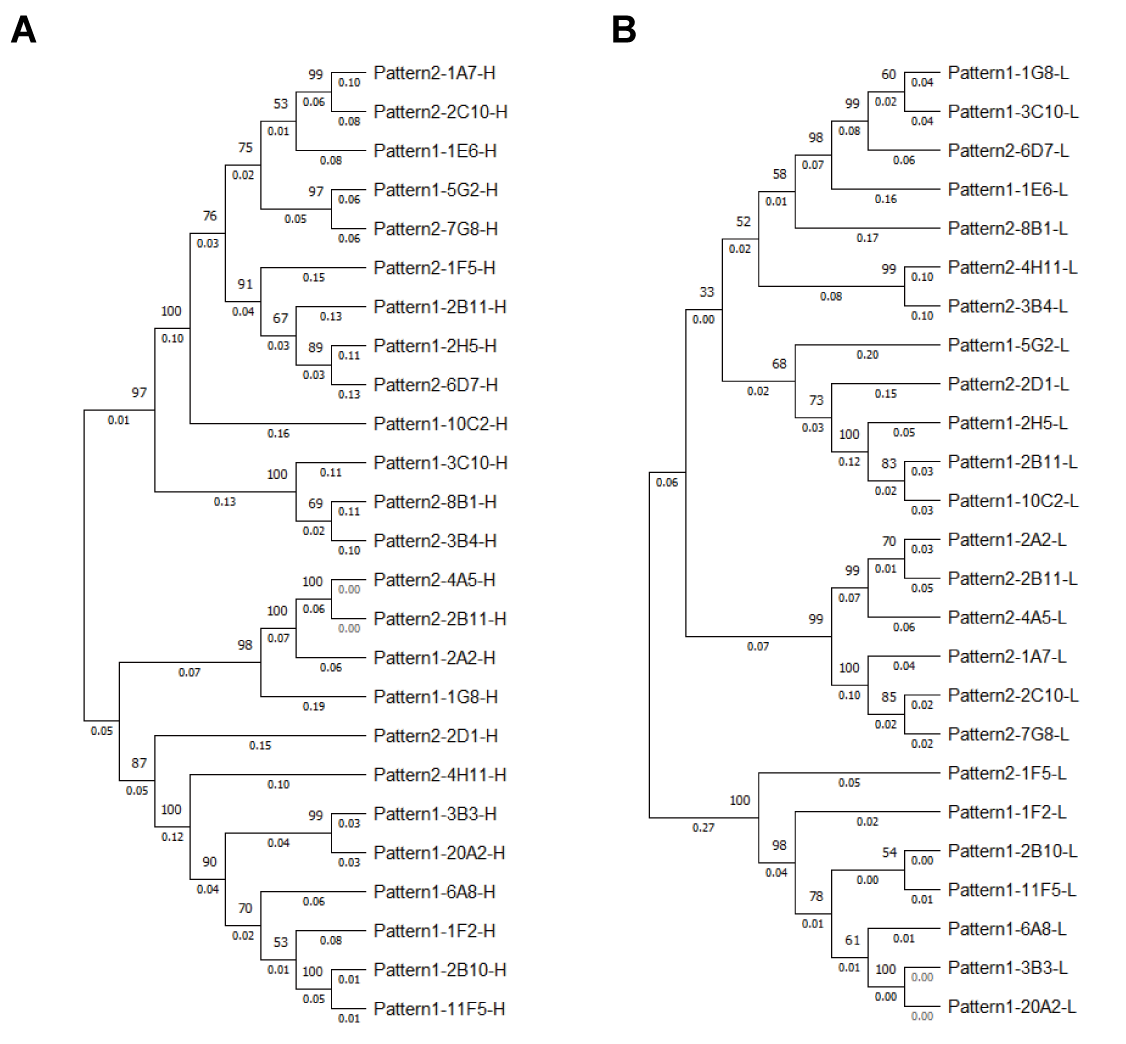


**Fig. S8.** The phylogenetic trees of the variable region of heavy chains (A) and light chains (B) for two patterns antibodies. The phylogenetic trees were developed by the Neighbor-Joining method using the MEGA software. P1, Pattern 1 antibodies; P2, Pattern 2 antibodies; A, the variable region of heavy chain; B, the variable region of light chain.


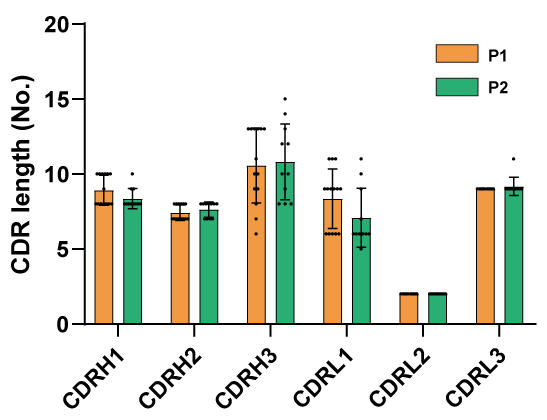


**Fig. S9.** The CDR length analysis of pattern 1 and pattern 2 antibodies. The CDRs of antibodies were annotated by the method of IMGT using abYsis website (http://www.abysis.org/abysis/index.html). P1, Pattern 1 antibodies; P2, Pattern 2 antibodies.


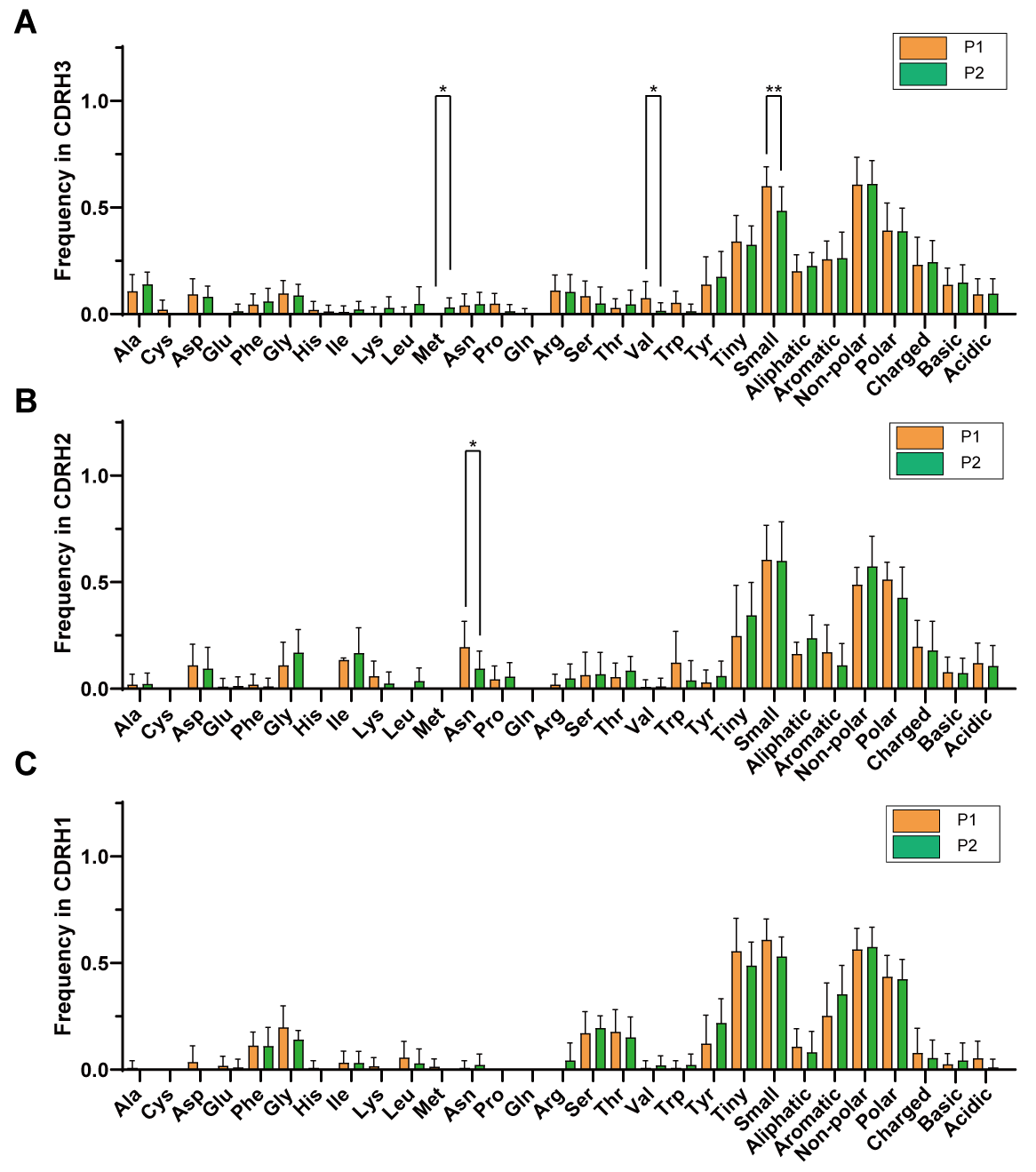


**Fig. S10.** The residue analysis for CDRH3 (A), CDRH2 (B) and CDRH1 (C) of pattern 1 and pattern 2 antibodies. To determine residue composition of CDRs, EMBOSS Pepstats was used (https://www.ebi.ac.uk/Tools/seqstats/emboss_pepstats/). P1, Pattern 1 antibodies; P2, Pattern 2 antibodies. Asterisks indicate significant difference between each two groups using two-tailed Mann-Whitney test (*p < 0.05; **p < 0.01; ***p < 0.001). The categories of residues were shown in Table S7.


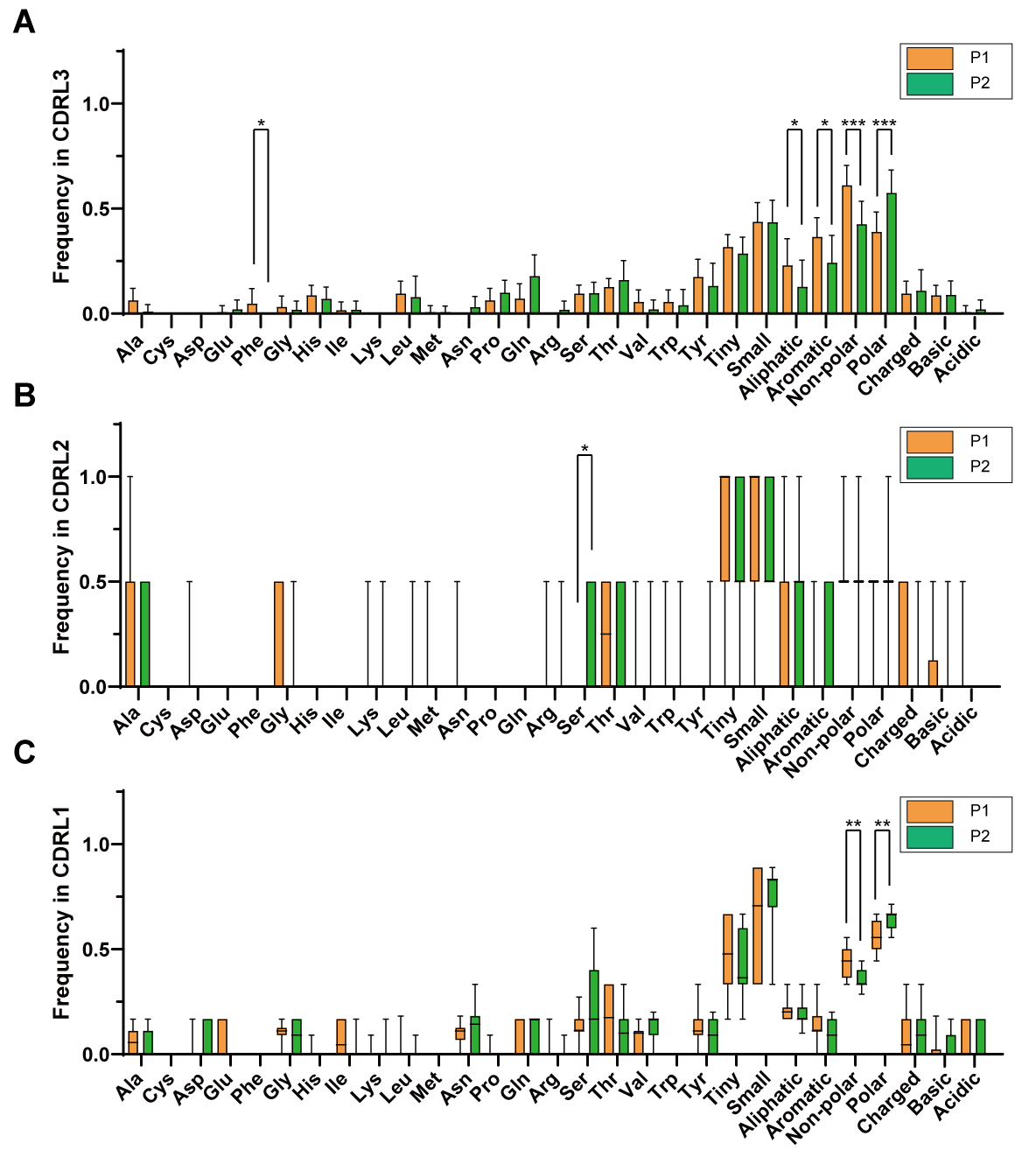


**Fig. S11.** The residue analysis for CDRL3 (A), CDRL2 (B) and CDRL1 (C) of pattern 1 and pattern 2 antibodies. To determine residue composition of CDRs, EMBOSS Pepstats was used (https://www.ebi.ac.uk/Tools/seqstats/emboss_pepstats/). P1, Pattern 1 antibodies; P2, Pattern 2 antibodies. Asterisks indicate significant difference between each two groups using two-tailed Mann-Whitney test (*p < 0.05; **p < 0.01; ***p < 0.001). The categories of residues were shown in Table S7.


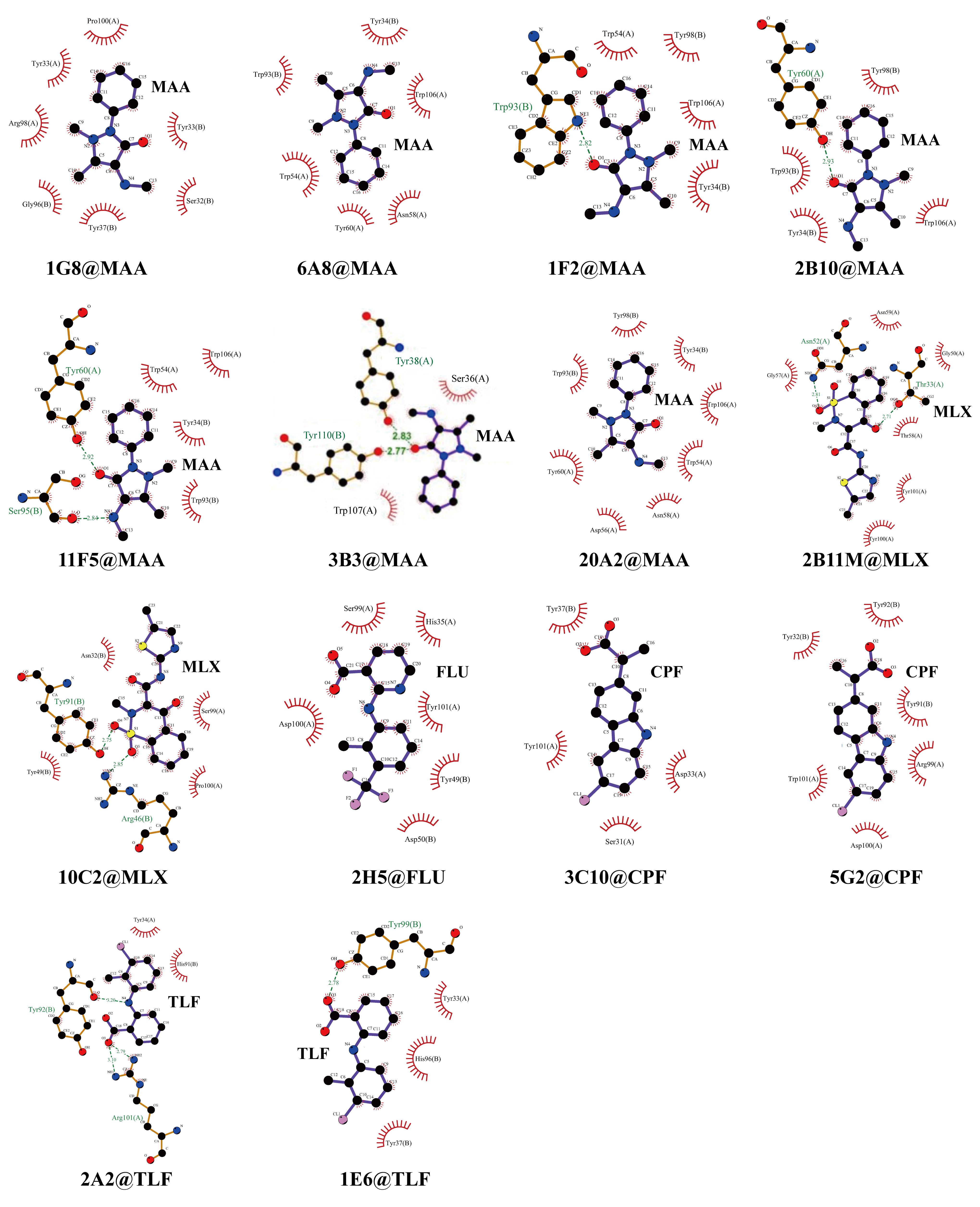


**Fig. S12.** The 2D interaction of Pattern 1 antibodies with their ligands. The 2D interactions were generated from the LigPlot; green dotted line represents the H-bond of the ligand with the amino acid of the antibody represented in green. Red spikes on the arcs show the hydrophobic interaction with the amino acids presented in black.


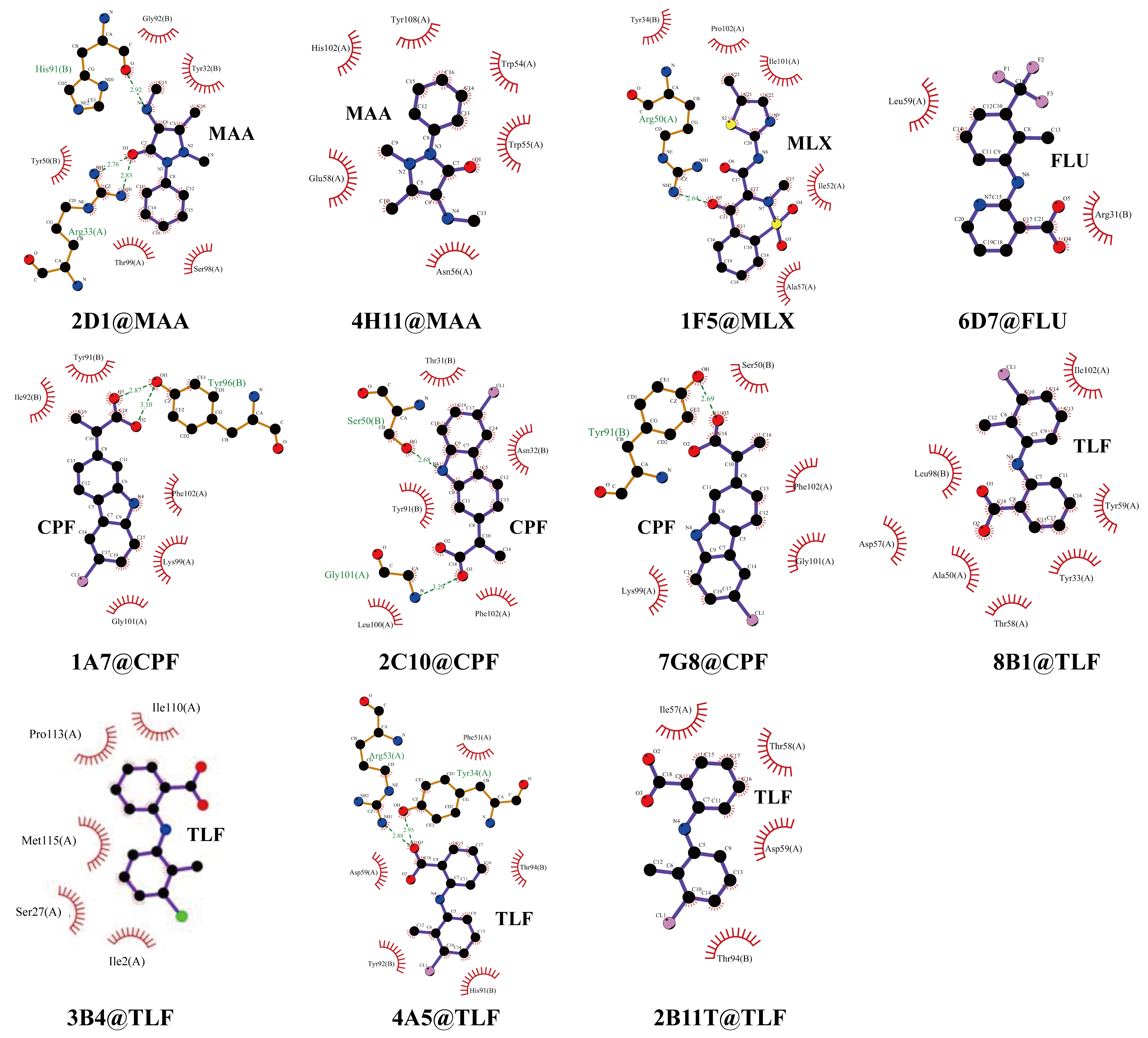


**Fig. S13.** The 2D interaction of Pattern 2 antibodies with their ligands. The 2D interactions were generated from the LigPlot; green dotted line represents the H-bond of the ligand with the amino acid of the antibody represented in green. Red spikes on the arcs show the hydrophobic interaction with the amino acids presented in black.


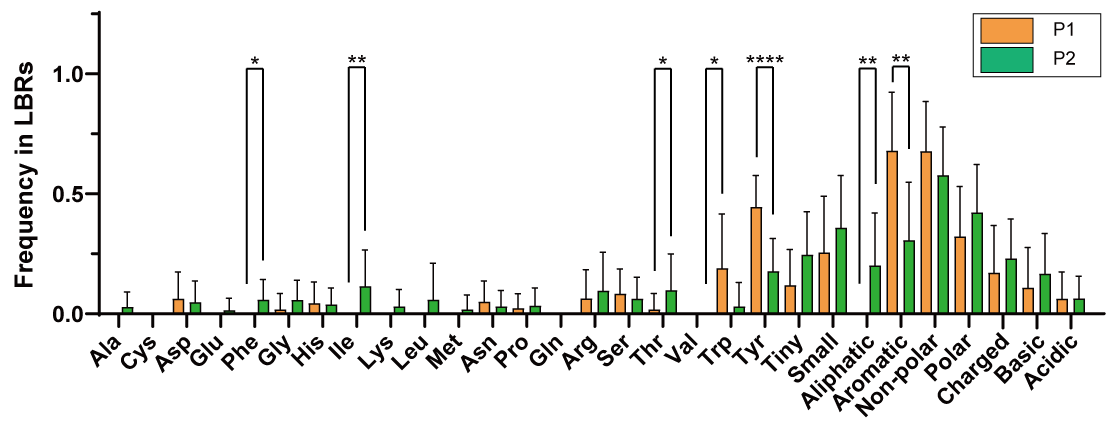


**Fig. S14.** The residue analysis of ligand-binding residues (LBRs) for pattern 1 and pattern 2 antibodies. To determine residue composition of CDRs, EMBOSS Pepstats was used (https://www.ebi.ac.uk/Tools/seqstats/emboss_pepstats/). P1, Pattern 1 antibodies; P2, Pattern 2 antibodies. Asterisks indicate significant difference between each two groups using two-tailed Mann-Whitney test (*p < 0.05; **p < 0.01; ***p < 0.001; ****p < 0.0001). The categories of residues were shown in Table S7.


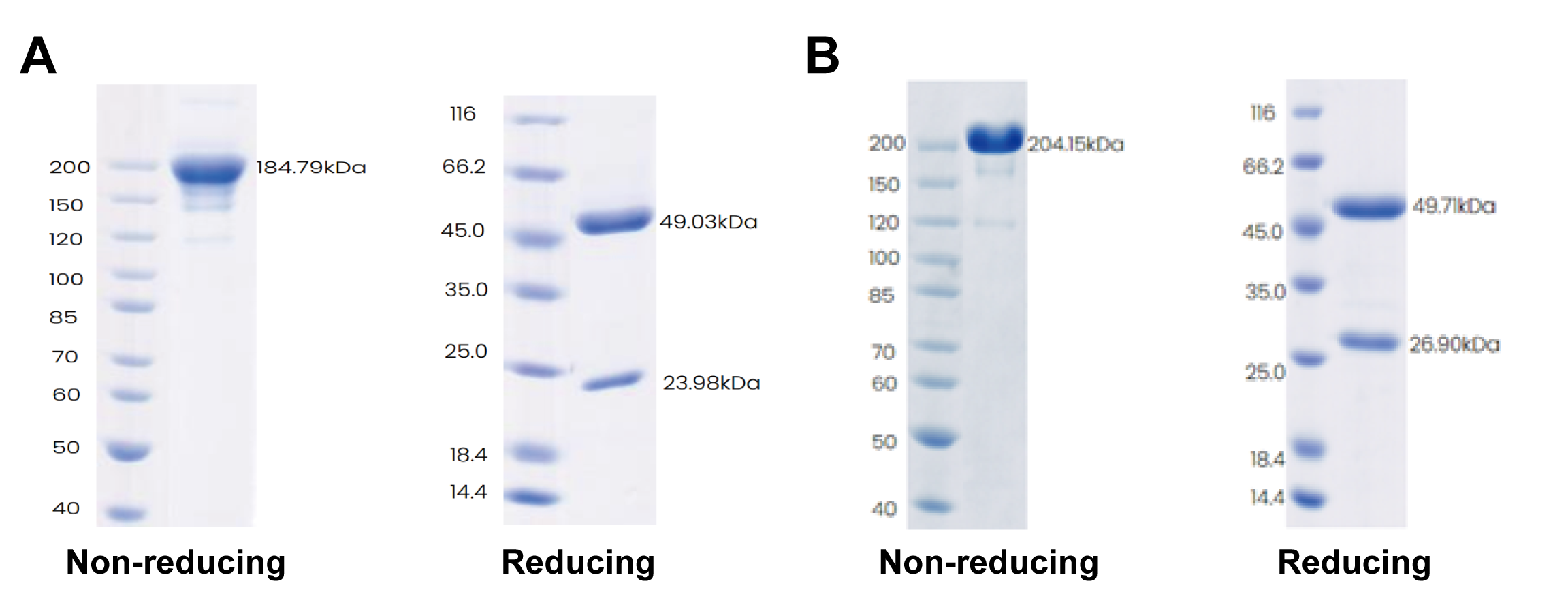


**Fig. S15.** The non-reducing and reducing polyacrylamide gel electrophoresis (PAGE) of 3B3-GL (A) and 3B4-GL (B). The results show that the molecular weights of whole antibodies, heavy chains and light chains3B3-GL and 3B4-GL were right.


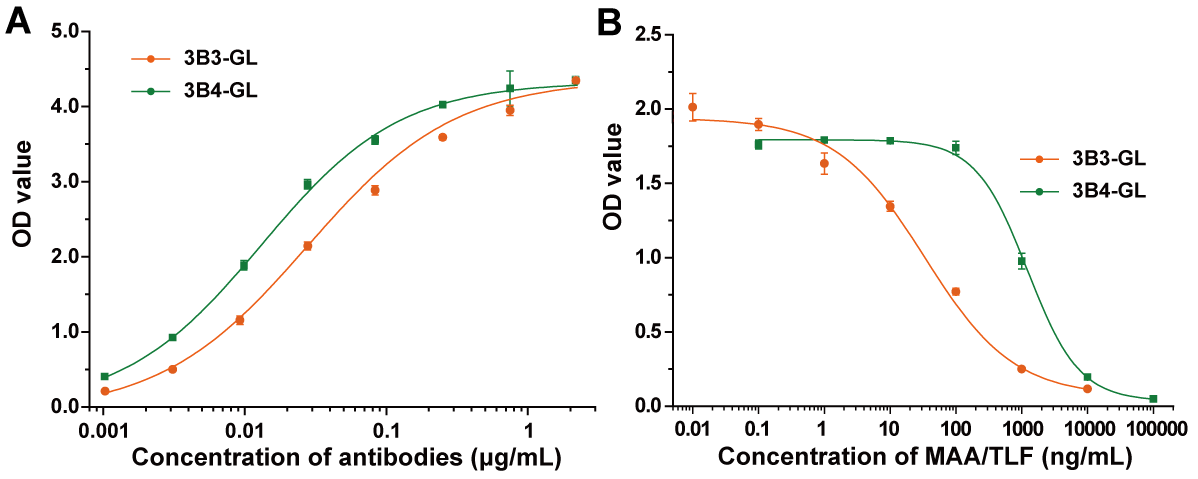


**Fig. S16.** The antigen-recognition properties of 3B3-GL and 3B4-GL were verified by antibody dilution curves (A) and competitive inhibition curves (B).

**Table S1.** The working concentrations of antibodies and coating antigen used in this work.

| Antibodies | Concentrations (ng/mL) | Coating antigens | Concentrations (ng/mL) |
| --- | --- | --- | --- |
| 1G8 | 74.1 | MAA-1-OVA | 24.7 |
| 6A8 | 24.7 | MAA-1-OVA | 8.2 |
| 1F2 | 24.7 | MAA-3-OVA | 8.2 |
| 2B10 | 2000.0 | MAA-3-OVA | 74.1 |
| 11F5 | 666.7 | MAA-3-OVA | 8.2 |
| 3B3 | 24.7 | MAA-2-OVA | 8.2 |
| 20A2 | 24.7 | MAA-2-OVA | 24.7 |
| 2B11M | 24.7 | MLX-1-OVA | 8.2 |
| 10C2 | 222.2 | MLX-2-OVA | 222.2 |
| 2H5 | 222.2 | FLU-OVA | 74.1 |
| 3C10 | 222.2 | CPF-OVA | 74.1 |
| 5G2 | 74.1 | CPF-OVA | 8.2 |
| 2A2 | 24.7 | TLF-OVA | 8.2 |
| 1E6 | 74.1 | TLF-OVA | 24.7 |
| 2D1 | 24.7 | MAA-3-OVA | 222.2 |
| 4H11 | 2000.0 | MAA-3-OVA | 8.2 |
| 1F5 | 24.7 | MAA-3-OVA | 8.2 |
| 6D7 | 24.7 | FLU-OVA | 8.2 |
| 1A7 | 74.1 | CPF-OVA | 24.7 |
| 2C10 | 74.1 | CPF-OVA | 24.7 |
| 7G8 | 24.7 | CPF-OVA | 24.7 |
| 8B1 | 24.7 | TLF-OVA | 8.2 |
| 3B4 | 24.7 | TLF-OVA | 24.7 |
| 4A5 | 74.1 | TLF-OVA | 24.7 |
| 2B11T | 74.1 | TLF-OVA | 24.7 |

**Table S2.** The methanol effect of reported antibodies to chemical compounds in methanol.

| Methanol effect patterns | Antibody names | Antibody origins | Analytes | Measurement methods | Position of the tolerance curves | References |
| --- | --- | --- | --- | --- | --- | --- |
| Pattern 1 | Nb26 | Alpaca | Aflatoxin B1 | ELISA | Figure S2C | Yan et al., 2022 ^1^ |
| Pattern 1 | Nb-B15 | Alpaca | Ustilaginoidins | ELISA | Figure 7A | W. Wang et al., 2022 ^2^ |
| Pattern 1 | 9A6 | Mouse | Chloramphenicol | ELISA | Figure S9G | Li et al., 2022 ^3^ |
| Pattern 1 | RmAb3 | Rabbit | Chloramphenicol | ELISA | Figure 4A | Li et al., 2022 ^4^ |
| Pattern 1 | D12E2 | Mouse | Aflatoxin | ELISA | Figure 2A | Ertekin et al., 2019 ^5^ |
| Pattern 1 | D3E4 | Mouse | Aflatoxin | ELISA | Figure 2A | Ertekin et al., 2019 ^5^ |
| Pattern 1 | Nb28 | Alpaca | Ochratoxin A | ELISA | Figure S1A | Sun et al., 2018 ^6^ |
| Pattern 1 | mAb | Mouse | α-Cyano Pyrethroid | CLEIA | Figure 2 | Taheri et al., 2016 ^7^ |
| Pattern 2 | B3G2 | Alpaca | iso-tenuazonic acid | ELISA | Figure 3D | F. Wang et al., 2022 ^8^ |
| Pattern 2 | 3E2 | Mouse | Phenylethanolamine A | ELISA | Figure 4C | Liu et al., 2022 ^9^ |
| Pattern 2 | Nb316 | Alpaca | Carbofuran | ELISA | Figure 4C | Zhang et al., 2019 ^10^ |
| Pattern 2 | 2B11 | Mouse | Aflatoxin | ELISA | Figure 2A | Ertekin et al., 2019 ^5^ |
| Pattern 2 | B5 | Mouse | Aflatoxin B1 | ELISA | Figure 5A | He et al., 2014 ^11^ |
| Pattern 2 | mAb | Mouse | Organophosphorus pesticides | CLEIA | Figure 1 | Xu et al., 2012 ^12^ |
| Pattern 2 | 3A2 | Mouse | Imidazolinone herbicides | ELISA | Figure 6 | Chin et al., 2002 ^13^ |
| Pattern 2 | OA8-2 | Mouse | Okadaic acid | ELISA | Table 1 | Matsuura et al., 1993 ^14^ |

ELISA, enzyme-linked immunosorbent assay; CLEIA, chemiluminescence enzyme immunoassay.

**Table S3.** The distribution of ligand-specific antibodies in Pattern 1 and Pattern 2 antibodies and the physicochemical properties of ligand.

| Ligands | Number of Pattern 1 antibodies | Number of Pattern 2 antibodies | Ratio | cLogP | Polar Surface Area (Å²) | Hydrogen Bond Donor | Hydrogen Bond Acceptor | MW (g/mol) | Rotatable Bond | Complexity |
| --- | --- | --- | --- | --- | --- | --- | --- | --- | --- | --- |
| MAA | 7 | 2 | 3.5 | 0.8 | 35.6 | 1 | 3 | 217.27 | 2 | 318 |
| MLX | 2 | 1 | 2 | 3 | 136 | 2 | 7 | 351.4 | 2 | 628 |
| CPF | 2 | 3 | 0.67 | 4 | 53.1 | 2 | 2 | 273.71 | 2 | 362 |
| FLU | 1 | 1 | 1 | 4.1 | 62.2 | 2 | 7 | 296.24 | 3 | 376 |
| TLF | 2 | 4 | 0.5 | 5.2 | 49.3 | 2 | 3 | 261.7 | 3 | 298 |

The physicochemical properties of ligand were computed by PubChem.

**Table S4.** The heavy chain and light chain isotypes of 25 antibodies to NSAIDs.

| Methanol effect patterns | Antibodies | Heavy chains | Light chains | Ligands |
| --- | --- | --- | --- | --- |
| Pattern 1 | 1G8 | IgG1 | Kappa | MAA |
| Pattern 1 | 6A8 | IgG1 | Lambda | MAA |
| Pattern 1 | 1F2 | IgG1 | Lambda | MAA |
| Pattern 1 | 2B10 | IgG1 | Lambda | MAA |
| Pattern 1 | 11F5 | IgG1 | Lambda | MAA |
| Pattern 1 | 3B3 | IgG1 | Lambda | MAA |
| Pattern 1 | 20A2 | IgG1 | Lambda | MAA |
| Pattern 1 | 2B11M | IgG1 | Lambda | MLX |
| Pattern 1 | 10C2 | IgG1 | Kappa | MLX |
| Pattern 1 | 2H5 | IgG1 | Kappa | FLU |
| Pattern 1 | 3C10 | IgG1 | Kappa | CPF |
| Pattern 1 | 5G2 | IgG2a | Kappa | CPF |
| Pattern 1 | 2A2 | IgG1 | Kappa | TLF |
| Pattern 1 | 1E6 | IgM | Kappa | TLF |
| Pattern 2 | 2D1 | IgG1 | Kappa | MAA |
| Pattern 2 | 4H11 | IgG2b | Kappa | MAA |
| Pattern 2 | 1F5 | IgG1 | Kappa | MLX |
| Pattern 2 | 6D7 | IgG1 | Kappa | FLU |
| Pattern 2 | 1A7 | IgG1 | Kappa | CPF |
| Pattern 2 | 2C10 | IgG2a | Kappa | CPF |
| Pattern 2 | 7G8 | IgG1 | Kappa | CPF |
| Pattern 2 | 8B1 | IgG1 | Kappa | TLF |
| Pattern 2 | 3B4 | IgG1 | Kappa | TLF |
| Pattern 2 | 4A5 | IgG1 | Kappa | TLF |
| Pattern 2 | 2B11T | IgG1 | Kappa | TLF |

**Table S5.** The sequence identity analysis of heavy chains of 25 NSAIDs antibodies.


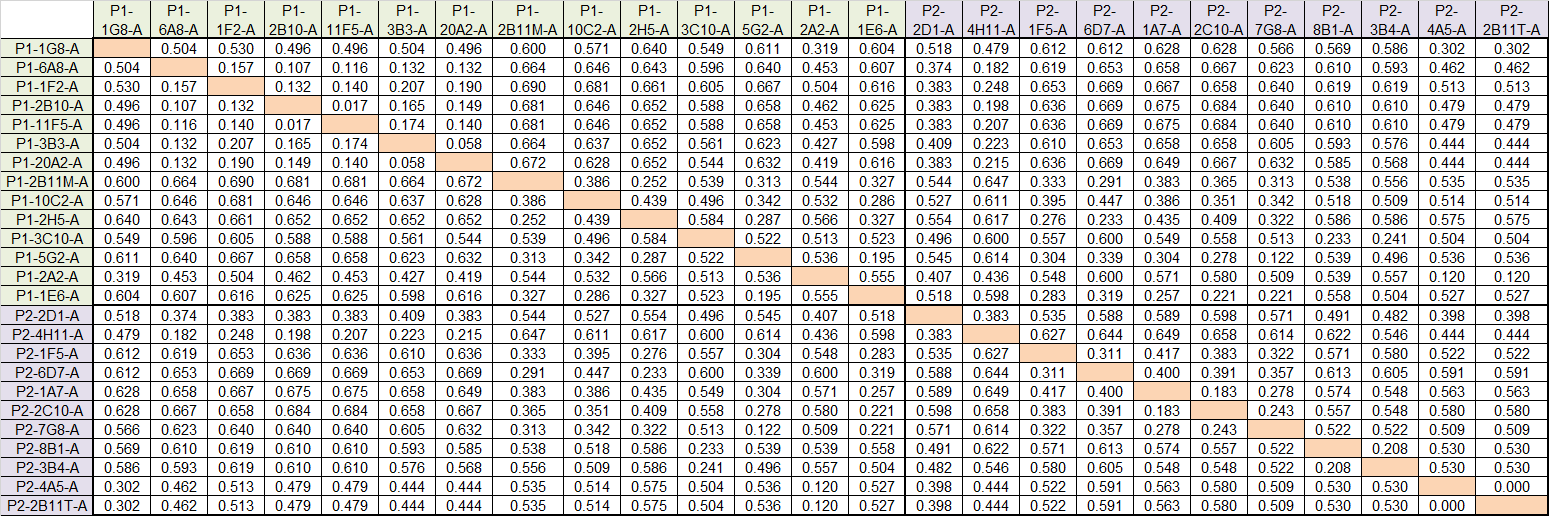


The multiple sequence alignment of the variable regions of heavy chain was conducted by ClustalW method using the MEGA. Then, the sequence identity was calculated by the pairwise distance method. P1, Pattern 1 antibodies; P2, Pattern 2 antibodies; A, the variable region of heavy chain.

**Table S6.** The sequence identity analysis of light chains of 25 NSAIDs antibodies.


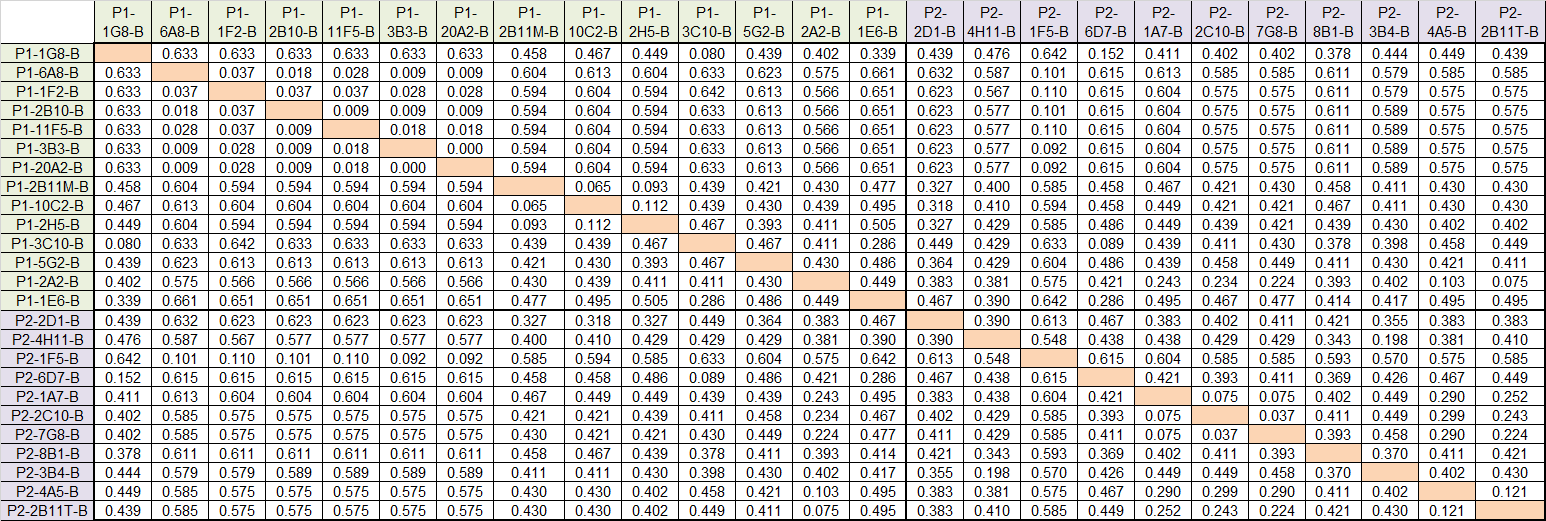


The multiple sequence alignment of the variable regions of heavy chain was conducted by ClustalW method using the MEGA. Then, the sequence identity was calculated by the pairwise distance method. P1, Pattern 1 antibodies; P2, Pattern 2 antibodies; B, the variable region of light chain.

**Table S7.** The categories of residues used in this study.

| Category | Residues |
| --- | --- |
| Tiny | Ala, Cys, Gly, Ser, Thr |
| Small | Ala, Asn, Asp, Cys, Gly, Pro, Ser, Thr, Val |
| Aliphatic | Ala, Ile, Leu, Val |
| Aromatic | Phe, His, Trp, Yyr |
| Non-polar | Ala, Cys, Phe, GLy, Ile, Leu, Met, Pro, Val, Trp, Tyr |
| Polar | Asp, Glu, His, Lys, Asn, Gln, Arg, Ser, Thr |
| Charged | Asn, Asp, Gln, Glu, His, Lys, Arg |
| Basic | His, Lys, Arg |
| Acidic | Asn, Asp, Gln, Glu |

**5. References:**

(1) Yan, T.; Zhu, J.; Li, Y.; He, T.; Yang, Y.; Liu, M. Development of a Biotinylated Nanobody for Sensitive Detection of Aflatoxin B1 in Cereal via ELISA. *Talanta* **2022**, *239*, 123125. https://doi.org/10.1016/j.talanta.2021.123125.

(2) Wang, W.; Gu, G.; Yin, R.; Fu, J.; Jing, M.; Shen, Z.; Lai, D.; Wang, B.; Zhou, L. A Nanobody-Based Immunoassay for Detection of Ustilaginoidins in Rice Samples. *Toxins* **2022**, *14* (10), 659. https://doi.org/10.3390/toxins14100659.

(3) Li, Y.; Li, P.; Ke, Y.; Yu, X.; Yu, W.; Wen, K.; Shen, J.; Wang, Z. Monoclonal Antibody Discovery Based on Precise Selection of Single Transgenic Hybridomas with an On-Cell-Surface and Antigen-Specific Anchor. *ACS Appl. Mater. Interfaces* **2022**, *14* (15), 17128–17141. https://doi.org/10.1021/acsami.2c02299.

(4) Li, Y.; Li, P.; Ke, Y.; Yu, X.; Yu, W.; Wen, K.; Shen, J.; Wang, Z. A Rare Monoclonal Antibody Discovery Based on Indirect Competitive Screening of a Single Hapten-Specific Rabbit Antibody Secreting Cell. *Analyst* **2022**, *147* (13), 2942–2952. https://doi.org/10.1039/D2AN00678B.

(5) Ertekin, Ö.; Kaymak, T.; Pirinçci, Ş. Ş.; Akçael, E.; Öztürk, S. Aflatoxin-Specific Monoclonal Antibody Selection for Immunoaffinity Column Development. *BioTechniques* **2019**, *66* (6), 261–268. https://doi.org/10.2144/btn-2018-0143.

(6) Sun, Z.; Lv, J.; Liu, X.; Tang, Z.; Wang, X.; Xu, Y.; Hammock, B. D. Development of a Nanobody-AviTag Fusion Protein and Its Application in a Streptavidin–Biotin-Amplified Enzyme-Linked Immunosorbent Assay for Ochratoxin A in Cereal. *Anal. Chem.* **2018**, *90* (17), 10628–10634. https://doi.org/10.1021/acs.analchem.8b03085.

(7) Taheri, N.; Lan, M.; Wei, P.; Liu, R.; Gui, W.; Guo, Y.; Zhu, G. Chemiluminescent Enzyme Immunoassay for Rapid Detection of Three α-Cyano Pyrethroid Residues in Agricultural Products. *Food Anal. Methods* **2016**, *9* (10), 2896–2905. https://doi.org/10.1007/s12161-016-0482-x.

(8) Wang, F.; Yang, Y.-Y.; Wan, D.-B.; Li, J.-D.; Liang, Y.-F.; Li, Z.-F.; Shen, Y.-D.; Xu, Z.-L.; Yang, J.-Y.; Wang, H.; Gettemans, J.; Hammock, B. D.; Sun, Y.-M. Nanobodies for Accurate Recognition of Iso-Tenuazonic Acid and Development of Sensitive Immunoassay for Contaminant Detection in Foods. *Food Control* **2022**, *136*, 108835. https://doi.org/10.1016/j.foodcont.2022.108835.

(9) Liu, M.; Bai, Y.; Dou, L.; Kong, Y.; Wang, Z.; Wen, K.; Shen, J. A Highly Salt-Tolerant Monoclonal Antibody-Based Enzyme-Linked Immunosorbent Assay for the Rapid Detection of Phenylethanolamine A in Urine. *Food and Agricultural Immunology* **2022**, *33* (1), 575–587. https://doi.org/10.1080/09540105.2022.2084043.

(10) Zhang, J.; Wang, Y.; Dong, J.; Yang, J.; Zhang, Y.; Wang, F.; Si, R.; Xu, Z.; Wang, H.; Xiao, Z.; Shen, Y. Development of a Simple Pretreatment Immunoassay Based on an Organic Solvent-Tolerant Nanobody for the Detection of Carbofuran in Vegetable and Fruit Samples. *Biomolecules* **2019**, *9* (10), 576. https://doi.org/10.3390/biom9100576.

(11) He, T.; Wang, Y.; Li, P.; Zhang, Q.; Lei, J.; Zhang, Z.; Ding, X.; Zhou, H.; Zhang, W. Nanobody-Based Enzyme Immunoassay for Aflatoxin in Agro-Products with High Tolerance to Cosolvent Methanol. *Anal. Chem.* **2014**, *86* (17), 8873–8880. https://doi.org/10.1021/ac502390c.

(12) Xu, Z.-L.; Sun, W.-J.; Yang, J.-Y.; Jiang, Y.-M.; Campbell, K.; Shen, Y.-D.; Lei, H.-T.; Zeng, D.-P.; Wang, H.; Sun, Y.-M. Development of a Solid-Phase Extraction Coupling Chemiluminescent Enzyme Immunoassay for Determination of Organophosphorus Pesticides in Environmental Water Samples. *J. Agric. Food Chem.* **2012**, *60* (9), 2069–2075. https://doi.org/10.1021/jf300225b.

(13) Chin, T. E.; Wong, R. B.; Pont, J. L.; Karu, A. E. Haptens and Monoclonal Antibodies for Immunoassay of Imidazolinone Herbicides. *J. Agric. Food Chem.* **2002**, *50* (12), 3380–3389. https://doi.org/10.1021/jf011481o.

(14) Matsuura, S.; Hamano, Y.; Kita, H.; Takagaki, Y. Preparation of Mouse Monoclonal Antibodies to Okadaic Acid and Their Binding Activity in Organic Solvents. *J Biochem* **1993**, *114* (2), 273–278. https://doi.org/10.1093/oxfordjournals.jbchem.a124166.
